# Characterization of the Ohmyungsamycin Biosynthetic Pathway and Generation of Derivatives with Improved Antituberculosis Activity

**DOI:** 10.3390/biom9110672

**Published:** 2019-10-30

**Authors:** Eunji Kim, Yern-Hyerk Shin, Tae Ho Kim, Woong Sub Byun, Jinsheng Cui, Young Eun Du, Hyung-Ju Lim, Myoung Chong Song, An Sung Kwon, Sang Hyeon Kang, Jongheon Shin, Sang Kook Lee, Jichan Jang, Dong-Chan Oh, Yeo Joon Yoon

**Affiliations:** 1 Department of Chemistry and Nanoscience, Ewha Womans University, Seoul 03760, Korea; ejkim0618@ewha.ac.kr (E.K.); smch517@ewha.ac.kr (M.C.S.); 2 Natural Products Research Institute, College of Pharmacy, Seoul National University, Seoul 08826, Korea; itsue00@snu.ac.kr (Y.-H.S.); sky_magic@naver.com (W.S.B.); cuijs@snu.ac.kr (J.C.); dye0302@snu.ac.kr (Y.E.D.); limju012@snu.ac.kr (H.-J.L.); shinj@snu.ac.kr (J.S.); sklee61@snu.ac.kr (S.K.L.); 3 Division of Applied Life Science (BK21plus Program), Gyeongsang National University, Jinju 52828, Korea; taeho12349@gmail.com (T.H.K.); jichanjang@gnu.ac.kr (J.J.); 4 iNtRON Biotechnology, Inc., Seongnam-si, Gyeonggi-do 13202, Korea; kwon4053@intron.co.kr (A.S.K.); kangsh0403@naver.com (S.H.K.)

**Keywords:** ohmyungsamycin, marine natural product, nonribosomal peptide synthetase, biosynthetic gene cluster, antituberculosis activity

## Abstract

The cyclic depsipeptides ohmyungsamycin (OMS) A (**1**) and B (**2**), isolated from the marine-derived *Streptomyces* sp. SNJ042, contain two non-proteinogenic amino acid residues, *β*-hydroxy-l-phenylalanine (*β*-hydroxy-l-Phe) and 4-methoxy-l-tryptophan (4-methoxy-l-Trp). Draft genome sequencing of *Streptomyces* sp. SNJ042 revealed the OMS biosynthetic gene cluster consisting of a nonribosomal peptide synthetase (NRPS) gene and three genes for amino acid modification. By gene inactivation and analysis of the accumulated products, we found that OhmL, encoding a P450 gene, is an l-Phe *β*-hydroxylase. Furthermore, OhmK, encoding a Trp 2,3-dioxygenase homolog, and OhmJ, encoding an *O*-methyltransferase, are suggested to be involved in hydroxylation and *O*-methylation reactions, respectively, in the biosynthesis of 4-methoxy-l-Trp. In addition, the antiproliferative and antituberculosis activities of the OMS derivatives dehydroxy-OMS A (**4**) and demethoxy-OMS A (**6**) obtained from the mutant strains were evaluated in vitro. Interestingly, dehydroxy-OMS A (**4**) displayed significantly improved antituberculosis activity and decreased cytotoxicity compared to wild-type OMS A.

## 1. Introduction

**Figure 1:**
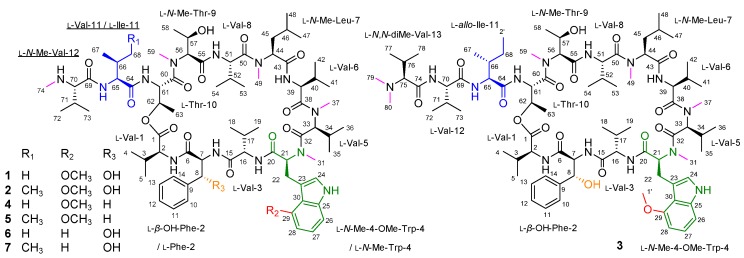
Structures of ohmyungsamycin (OMS) A (**1**), OMS B (**2**), ecumicin (**3**), and OMS derivatives (**4**–**7**).

The cyclic depsipeptides ohmyungsamycin A (OMS A) (**1**) and OMS B (**2**) were isolated from a marine-derived *Streptomyces* sp. strain and showed inhibitory effects towards diverse cancer cells and bacteria [1], as well as antituberculosis activity against *Mycobacterium tuberculosis* [2,3]. The structures of OMSs A (**1**) and B (**2**) have been determined predominantly by spectroscopic analysis [1], and the structure of OMS B has recently been updated through total synthesis (Figure 1): l-Val-11 and l-*N*,*N*-dimethyl-Val-12 were revised to l-Ile-11 and l-*N*-methyl-Val-12 (underlined in Figure 1), respectively [3]. The OMSs are composed of 12 amino acid units, including two non-proteinogenic and four *N*-methylated amino acids [1,3]. Ecumicin (**3**), another antitubercular peptide, is a tridecapeptide with an identical cyclic peptide core as that of OMSs (Figure 1) [4]. Both OMSs and ecumicin include the same two non-proteinogenic amino acid residues, *β*-hydroxy-l-phenylalanine (*β*-hydroxy-l-Phe) and 4-methoxy-l-tryptophan (4-methoxy-l-Trp). Whereas *β*-hydroxylation of amino acids often occurs in the biosynthesis of several nonribosomal peptides (NRPs), 4-methoxy-l-Trp is rarely found in NRP biosynthesis [4,5].

The formation of *β*-hydroxylated amino acids in NRP compounds, such as skyllamycin [6,7,8], balhimycin [9], and echinomycin [10], is known to be catalyzed by cytochrome P450 enzymes (P450s) [11]. For example, a single P450 from the skyllamycin biosynthetic cluster (Sky32) is responsible for the *β*-hydroxylation of three amino acids in the biosynthesis of skyllamycin. Sky32 interacts with certain peptidyl carrier proteins (PCPs) to catalyze the hydroxylation of PCP-bound amino acids at the *β*-position [6,7,8]. The 23 conserved amino acid residues recognizing the PCP domains have been identified in such *trans*-interacting P450s involved in the hydroxylation of PCP-bound peptide chains (Figure S1) [6,9,11].

A diverse range of enzymatic modifications of the Trp moiety, including methylation, oxidation, chlorination, and prenylation, have been observed in the biosynthesis of natural products [12]. For example, cyclic peptides derived from marine organisms, such as discobahamins A/B [13], calyxamides A/B [14], keramamides [15], and orbiculamide A [16], contain the Trp moiety hydroxylated at position 5 of the indole (Figure S2A). The formation of 5-methoxy-l-Trp has been well characterized: Trp hydroxylase-1 converts l-Trp to 5-hydroxy-l-Trp, which is then methylated to 5-methoxy-l-Trp by hydroxyindole *O*-methyltransferase [17]. However, 4-methoxy-l-Trp is less common in nature, and its biosynthesis has remained obscure. To the best of our knowledge, besides OMSs and ecumicin, only argyrins, macrocyclic peptides produced by marine myxobacterium *Cystobacter* spp., possess the 4-methoxy-l-Trp moiety [5]. This unusual moiety is essential for the antiproteasomal and antibacterial activities of argyrins [18,19].

We report herein the identification of the gene cluster governing OMS biosynthesis from the *Streptomyces* sp. SNJ042 through draft genome sequencing. Further gene inactivation and complementation associated with bioinformatic analysis allowed the functional prediction of the enzymes involved in the biosynthesis of unusual amino acid moieties. In addition, the antiproliferative and antituberculosis activities of OMS derivatives (Figure 1) generated by deletion mutant strains were evaluated, providing a structure–activity relationship between the functional groups in the unusual amino acids and the bioactivity.

## 2. Results and Discussion

### 2.1. Identification of the OMS Biosynthetic Gene Cluster

**Figure 2:**
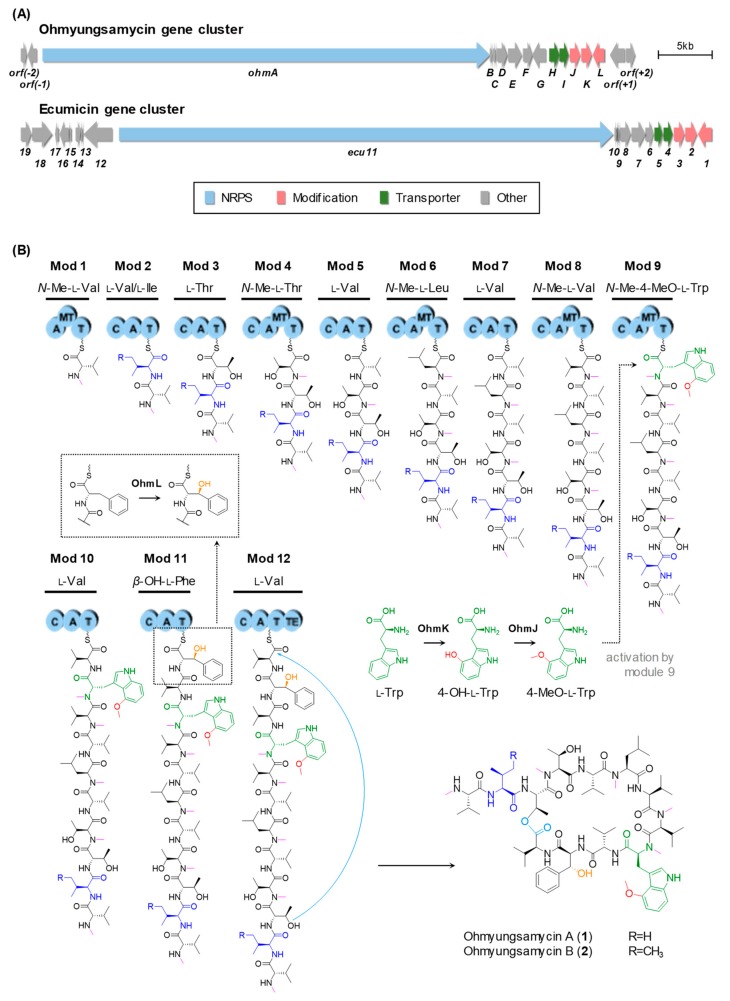
Proposed biosynthetic pathway for OMS A and OMS B. (**A**) Genetic organization of *ohm* and *ecu* gene clusters. (**B**) OMS modular NRP synthetase (NRPS) assembly line and putative biosynthetic pathway. A, adenylation domain; C, condensation domain; MT, *N*-methyltransferase domain; T, thiolation domain (also known as peptidyl carrier protein (PCP) domain); TE, thioesterase domain.

To identify the OMS biosynthetic gene cluster, draft genome sequencing of the *Streptomyces* sp. SNJ042 was carried out, and bioinformatic analysis using antiSMASH (antibiotics and Secondary Metabolite Analysis Shell) [20] revealed the presence of a modular NRP synthetase (NRPS) gene cluster as the candidate for the OMS gene cluster. We localized a DNA region of approximately 57 kb, designated as the *ohm* gene cluster, consisting of 16 open reading frames. It contains a 41.4 kb single *NRPS* gene encoding a total of 12 modules consistent with the proposed peptide structure, three genes related to amino acid modifications, two transport genes, and others (Table S1). This *ohm* cluster is highly homologous to the ecumicin gene cluster (*ecu*) [4] (GenBank: KJ144825.1), notably including the same amino acid modification genes encoding an *O*-methyltransferase (*O*-MT), a Trp 2,3-dioxygenase (TDO), and a P450 monooxygenase (Table S1). The genetic organization of the *ohm* and *ecu* gene clusters is described in Figure 2A.

The OMS peptide backbone is composed of 12 amino acids, as shown in Figure 2B. The NRPS OhmA encodes 12 modules containing the entire 41 domains. Each module includes a minimal NRPS module consisting of a condensation (C) domain, an adenylation (A) domain, and a PCP domain (C-A-PCP), except for the initiation module. Specificity prediction for almost all the A domains based on specificity-conferring sequences [21,22] coincided with the specificity of the postulated substrates, although an inconsistency was observed for the A domain of module 6 (A6 domain) (Table S2). Although the A6 domain was predicted to activate Phe or Trp, Leu should be activated by the A6 domain and incorporated according to the OMS structure. Moreover, in line with the presence of *N*-methylated amino acid units in the OMS scaffold, an additional modifying domain, an *N*-methyltransferase (*N*-MT) domain, is located in modules 1, 4, 6, 8, and 9. In addition, a thioesterase (TE) domain, which catalyzes the release of the mature peptide, is located at the C-terminus of module 12. Thus, this domain organization supports that OMS biosynthesis is achieved by sequential incorporation of 12 amino acids from l-Val at module 1 to another l-Val at module 12 and by the cyclization between l-Val and l-Thr units originated from modules 12 and 3, respectively (Figure 2B).

The structural difference between OMS A and OMS B is probably caused by the substrate promiscuity of the A domain of module 2 (A2 domain): the activation of l-Val by the A2 domain produces OMS A, while OMS B is biosynthesized through the incorporation of l-Ile by the A2 domain. Analysis of A domains in Ohm NRPS showed that the conserved amino acid-specific motifs of the A2 domain (DAYWGGAT) are distinct from those of other Val-specific A1, A5, A7, A8, A10, and A12 domains (DAYWWGGT) (Table S2). Through the comparison of such eight key residues of the substrate-binding pocket, which show the identity for substrate recognition [21,22], we could expect that the A2 domain has more relaxed substrate specificity than other Val-specific A domains and might activate both l-Val and l-Ile. Thus, it is plausible that the substrate utilization of module 2 determines whether OMS A or OMS B is produced.

### 2.2. Biosynthesis of the β-Hydroxy-l-Phe Moiety

**Figure 3:**
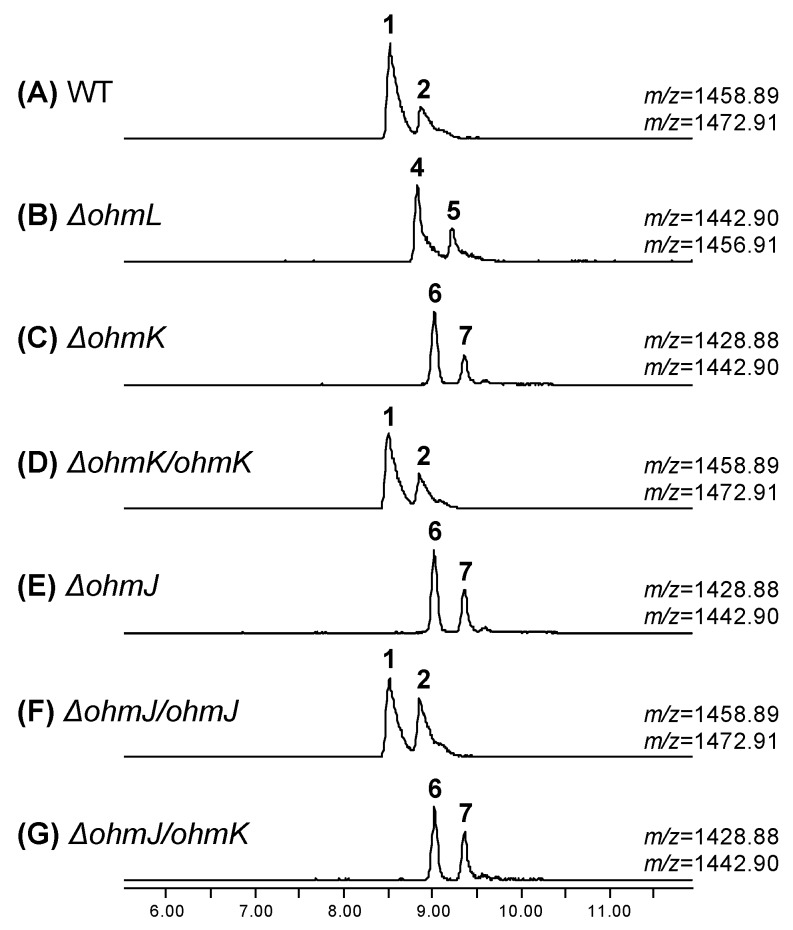
UPLC-qTOF-HR-MS analysis of the OMSs and derivatives produced by the wild-type and mutant strains of *Streptomyces* sp. SNJ042. Chromatograms are shown for selected *m/z* values of naturally occurring OMS A (**1**, [M+H]^+^ = 1458.89) and OMS B (**2**, [M+H]^+^ = 1472.91) and the derivatives produced by each mutant strain. (**A**) Chromatogram of OMS A (**1**) and OMS B (**2**) produced from the wild-type strain. (**B**) Chromatogram of dehydroxy-OMS A (**4**, [M+H]^+^ = 1442.90) and dehydroxy-OMS B (**5**, [M+H]^+^ = 1456.91) produced from the *ΔohmL* strain. (**C**) Chromatogram of demethoxy-OMS A (**6**, [M+H]^+^ = 1428.88) and demethoxy-OMS B (**7**, [M+H]^+^ = 1442.90) produced from the *ΔohmK* strain. (**D**) Chromatogram of OMS A (**1**) and OMS B (**2**) produced from the *ΔohmK/ohmK* strain. (**E**) Chromatogram of demethoxy-OMS A (**6**, [M+H]^+^ = 1428.88) and demethoxy-OMS B (**7**, [M+H]^+^ = 1442.90) produced from *ΔohmJ* strain. (**F**) Chromatogram of OMS A (**1**) and OMS B (**2**) produced from the *ΔohmJ/ohmJ* strain. (**G**) Chromatogram of demethoxy-OMS A (**6**, [M+H]^+^ = 1428.88) and demethoxy-OMS B (**7**, [M+H]^+^ = 1442.90) produced from *ΔohmJ/ohmK* strain.

Both OMS A and OMS B possess a *β*-hydroxy-l-Phe moiety, which is expected to be catalyzed by P450 OhmL. This enzyme shows similarity to Sky32 (54%) from the skyllamycin biosynthetic gene cluster [6,7,8], to OxyD (57%) from the balhimycin biosynthetic gene cluster [9], and to Ecm12 (52%) from the echinomycin biosynthetic gene cluster [10]. Moreover, OhmL possesses the consensus aminoacyl-PCP binding motif (Figure S1). To confirm the function of OhmL, the *ohmL* gene was inactivated by in-frame gene deletion (Figure S3). Two compounds corresponding to dehydroxy-OMS A (**4**) and dehydroxy-OMS B (**5**) were detected in the *ohmL* deletion mutant strain (*ΔohmL* strain), with *m*/*z* = 1442.90 and *m*/*z* = 1456.91, respectively, by UPLC–qTOF-HRMS analysis (Figure 3B). Compounds **4** and **5** were proposed to be the derivatives of OMS A (**1**) and OMS B (**2**), respectively, dehydroxylated at the *β*-position of the Phe moiety on the basis of the MS/MS fragmentation patterns (Figure S4A,B). These results indicate that OhmL is an l-Phe *β*-hydroxylase.

**Figure 4:**
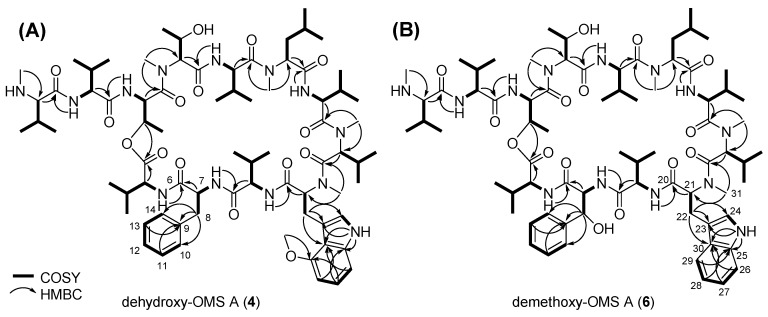
Key COSY and HMBC correlations for structure determination of (**A**) **4** and (**B**) **6**.

Dehydroxy-OMS A (**4**) is a major product of the *ΔohmL* strain, while OMS A (**1**) is a major product of the wild-type strain and exhibits more potent biological activity than OMS B (**2**) [3]. Hence, we decided to isolate and determine the structure of dehydroxy-OMS A (**4**). Dehydroxy-OMS A (**4**) was purified as a yellowish powder via consecutive chromatographic isolations. The molecular formula of **4** was established as C_75_H_119_N_13_O_15_ on the basis of high-resolution fast atom bombardment mass spectrometry (HR-FAB-MS, Figure S5) and NMR data (Figures S6–S12). The unsaturation number was calculated to be 23 on the basis of the molecular formula of **4**. By combinational analysis of the ^1^H and HSQC NMR data (Figures S6 and S10), eight amide protons (δ_H_ 11.80, 10.40, 10.31, 9.92, 9.46, 9.17, 8.08, and 8.04) and nine aromatic protons (δ_H_ 7.40 [2H], 7.34 [2H], 7.30, 7.27, 7.24, 7.19, and 6.68) of **4** in the downfield region were identified. In addition, six methyl groups substituted at heteroatoms were detected at δ_H_ 3.85, 3.71, 3.53, 3.20, 2.99, and 2.51. Careful analysis of the ^13^C NMR data (Figure S7) showed the existence of 12 carbonyl carbon signals at δ_C_ 175–168 (δ_C_ 174.6, 174.2, 173.6, 173.0, 172.8, 172.6, 171.7, 171.3, 170.6, 169.8, 169.5, and 168.1). Furthermore, 14 aromatic carbons and an oxygenated aromatic carbon were also observed at δ_C_ 155–99. Combined analysis of the 1D and 2D NMR data (Figures S6–S12), showed that **4** consists of amino acid units identical to those OMS A, apart from one unit: **4** possesses a Phe unit in its structure instead of the *β*-hydroxy-Phe in **1**. On the basis of consecutive COSY correlations between aromatic protons, H-10 (δ_H_ 7.34, d, *J* = 7.5 Hz), H-11 (δ_H_ 7.40, dd, *J* = 7.5, 7.0 Hz), H-12 (δ_H_ 7.24, dd, *J* = 7.0, 7.0 Hz), H-13 (δ_H_ 7.40, dd, *J* = 7.5, 7.0 Hz), and H-14 (δ_H_ 7.34, d, *J* = 7.5 Hz), and the coupling constant values between each proton, a partial structure of the Phe unit, C-10–C-11–C-12–C-13–C-14, could be deciphered. The ^1^H–^13^C HMBC correlations from H-11 and H-13 to C-9 (δ_C_ 139.3) verified the six-membered aromatic ring structure, and another HMBC correlation from H_2_-8 (δ_H_ 3.80 and 3.28) to C-9, C-10 (δ_C_ 129.8), and C-14 (δ_C_ 129.8) indicated that the CH_2_-8 was substituted at C-9. The CH_2_-8 could be traced to 7-NH (*δ*_H_ 10.40) by serial ^1^H–^1^H coupling correlations, and the HMBC correlation of H-7 (δ_H_ 5.49)/C-6 (δ_C_ 172.8) completed the Phe unit in **4**. The other amino acid units, namely, Val, *N*-methyl-Val, *N*-methyl-Leu, Thr, *N*-methyl-Thr, and *N*-methyl-4-methoxy-Trp, were identified by comparing their chemical shifts with those of **1** and by combined analysis of 1D and 2D NMR spectroscopic data (Table 1, Figure 4A) [1]. The absolute configurations of **4** could be assigned by comparing its CD spectrum with that of **1** (Figure S13).

### 2.3. Biosynthesis of the 4-Methoxy-l-Trp Moiety

We reasoned that the 4-methoxy-l-Trp moiety could be biosynthesized in two steps, i.e., hydroxylation followed by *O*-methylation at the 4-position of the indole ring. In the *ohm* gene cluster, we found only one P450 (OhmL), which was shown to be responsible for *β*-hydroxylation of a PCP-attached amino acid by gene deletion, as shown in Figure 3B. Therefore, we could expect that a putative TDO along with an *O*-MT, respectively encoded by *ohmK* and *ohmJ*, may catalyze the desired hydroxylation and methylation. OhmK and OhmJ show high similarity to Arg5 (59%) and Arg4 (72%), respectively, which are involved in the biosynthesis of argyrins [5]. Two genes homologous to *ohmK* and *ohmJ* were also found in the gene cluster of ecumicin, which possesses the same 4-methoxy-l-Trp moiety (Figure 2A).To verify the role of OhmK and OhmJ in the formation of the 4-methoxy-l-Trp moiety, *ohmK* and *ohmJ* were separately inactivated by in-frame deletion (Figure S3). The *ohmK* deletion mutant strain (*ΔohmK* strain) produced two expected products, demethoxy-OMS A (**6**) and demethoxy-OMS B (**7**), both of which lack a methoxy group on the Trp moiety (Figure 3C). They were observed with *m/z* = 1428.88 and *m/z* = 1442.90, and their structures were predicted by MS/MS analysis (Figure S4C). In addition, self-complementation of the *ohmK* gene in the *ΔohmK* mutant (*ΔohmK/ohmK* strain) restored the production of OMS A (**1**) and OMS B (**2**) (Figure 3D). Unexpectedly, the *ohmJ* deletion mutant (*ΔohmJ* strain) resulted in the production of compounds **6** and **7** (Figure 3E and Figure S4D), although we expected the production of OMS derivatives containing the 4-hydroxy-l-Trp moiety, owing to the absence of the *O*-MT function in the *ΔohmJ* mutant strain. Genetic complementation of *ohmJ* in the *ΔohmJ* mutant strain (*ΔohmJ/ohmJ* strain) restored the production of OMS A (**1**) and OMS B (**2**), whereas the expression of *ohmK* in the *ΔohmJ* mutant strain (*ΔohmJ/ohmK* strain) still only led to compounds **6** and **7** (Figure 3F,G) and not to the production of the OMS derivatives containing the 4-hydroxy-l-Trp moiety. Therefore, it is plausible that OhmK hydroxylates free l-Trp at the 4-position, and then OhmJ *O*-methylates 4-hydroxy-l-Trp, forming 4-methoxy-l-Trp, which is introduced into module 9 of the *ohm* NRPS assembly line. In this process, it is likely that 4-hydroxy-l-Trp could not be activated by the A9 domain, while l-Trp or 4-methoxy-l-Trp could be activated by the A9 domain and used as an extender unit in module 9 during OMS biosynthesis.

TDO generally catalyzes the oxidation of l-Trp to *N*-formyl-l-kynurenine in the biosynthesis of secondary metabolites, such as echinomycin [10], daptomycin [23], antimycin [24], and sibiromycin [25] (Figure S2B), in addition to primary metabolism. In contrast, in this study, it was suggested that the putative TDO homolog OhmK is involved in the hydroxylation of free Trp in OMS biosynthesis and is essential for the formation of 4-methoxy-l-Trp. Phylogenetic analysis revealed that OhmK, Ecu2, and Arg5 are positioned in the same clade, which is separated from the other TDOs mentioned above (Figure S14). Taken together, our results clearly show that OhmK is an l-Trp 4-hydroxylase.

The molecular formula of demethoxy-OMS A (**6**), which was isolated as a yellow powder, was determined as C_74_H_117_N_13_O_15_ by analysis of HR-FAB-MS data (Figure S15) along with ^1^H and ^13^C NMR spectra (Figures S16 and S17). Its degree of unsaturation was calculated as 23, on the basis of the molecular formula. The existences of 8 amide protons (δ_H_ 11.87, 10.29, 9.96, 9.73, 9.36, 9.26, 8.15, and 8.08) and 10 aromatic protons (δ_H_ 7.86, 7.65 [3H], 7.38 [3H], 7.35, and 7.24 [2H]) in **6** was confirmed on the basis of ^1^H NMR data (Figure S16). Additionally, protons for five *N*-methyl groups were detected in the 4.0–2.4 ppm region as singlet peaks. From the ^13^C NMR data (Figure S17), 12 carbonyl carbons (δ_C_ 174.4, 174.3, 173.5, 173.1, 172.7, 172.6, 171.6, 171.4, 170.6, 169.5, 169.4, and 168.0) of **6** were identified. The planar structure of **6** was determined via analysis of its 1D and 2D NMR spectra (Figures S16–S22). A series of COSY correlations (^3^*J*_H26H27_ = 7.5 Hz and ^3^*J*_H28H29_ = 7.5 Hz) among H-26 (δ_H_ 7.65), H-27 (δ_H_ 7.38), H-28 (δ_H_ 7.24), and H-29 (δ_H_ 7.86) showed C-26–C-27–C-28–C-29 connectivity, indicating the existence of an *ortho*-substituted aromatic ring. Three-bond HMBC correlations from H-26/H-28 to C-30 (δ_C_ 128.5) and from H-27/H-29 to C-25 (δ_C_ 137.8) were assigned to the six-membered aromatic ring structure. A COSY correlation between H-24 (δ_H_ 7.35) and 24-NH (δ_H_, 11.87) indicated C-24–NH connectivity, and ^1^H-^13^C HMBC correlations from 24-NH to C-23 (δ_C_ 113.1), C-25, and C-30 indicated an indole moiety. Linkage between C-21 and C-22 was confirmed via ^1^H–^1^H coupling between H-21 (δ_H_ 4.21) and H_2_-22 (δ_H_ 4.40 and 4.30). HMBC correlations from H_2_-22 to C-23/C-24 (δ_C_ 124.5)/C-30, from H-21 to C-20 (δ_C_ 169.4), and from H_3_-31 (δ_H_ 2.46) to C-21 (δ_C_ 70.7) confirmed the *N*-methyl-Trp unit in **6** instead of the *N*-methyl-4-methoxy-Trp in **1**. Similarly to **4**, the other amino acid units comprising **6** were determined by comparing the ^1^H and ^13^C chemical shifts with those for **1** and by spectroscopic analysis of the 1D and 2D NMR spectral data (Table 2, Figure 4B) [1]. The stereogenic centers in **6** were identified by comparative analysis of the CD spectral data for **1** and **6** (Figure S13).

### 2.4. Antituberculosis Activity

To test whether the OMS analogs have enhanced growth inhibitory activity against *M. tuberculosis* mc^2^ 6230, we conducted drug susceptibility tests with OMS A (**1**), dehydroxy-OMS A (**4**), and demethoxy-OMS A (**6**) using the resazurin microtiter assay (REMA). The growth inhibitory activities of the compounds were compared with those of isoniazid (INH) and ethambutol (EMB), which are currently used for tuberculosis treatment in the clinic. As shown in Table 3, all the tested compounds significantly decreased resazurin fluorescence in a concentration-dependent manner. Furthermore, all the OMS analogs showed much stronger activity than the reference compounds INH and EMB. Among the compounds tested, dehydroxy-OMS A (**4**) showed the strongest inhibitory activity, with a minimum inhibitory concentration of the compounds at which 50% of the isolates were inhibited (MIC_50_) of 4.9 nM in 7H9 medium, which was more than six times higher than that of OMS A. However, demethoxy-OMS A (**6**) exhibited an MIC_50_ approximately 1.3-fold higher than that of OMS A (**1**).

### 2.5. Antiproliferative Activity

OMS A was also reported to display cytotoxicity against human lung cancer A549, colon cancer HCT116, stomach cancer SNU638, liver cancer SK-HEP-1, and breast cancer MDA-MB-231 cell lines, which are major solid-cancer types, whereas it did not show antiproliferative effect against the normal lung fibroblast MRC-5 cell line [1]. Therefore, the antiproliferative activities of OMS A (**1**), dehydroxy-OMS A (**4**), and demethoxy-OMS A (**6**) were measured against these cell lines for comparison. As displayed in Table 3, demethoxy-OMS A (**6**) showed similar antiproliferative activities (IC_50_ values: 7.68–11.58 μM) to that of OMS A (IC_50_ values: 10.91–14.90 μM) against the tested cell lines. However, dehydroxy-OMS A (**4**) exhibited a relatively lower antiproliferative activity compared to OMS A and compound **6** toward human cancer cells. In addition, all the OMS analogs did not show cytotoxicity against the normal lung fibroblast cell line MRC5 (IC_50_ > 50 μM), suggesting a relatively specific cytotoxic activity against human cancer cells compared to normal cells.

## 3. Materials and Methods

### 3.1. General Experimental Procedures

Optical rotation data at 25 °C were acquired using a JASCO (Easton, MD, USA) P-2000 polarimeter with a 1 cm cell. Ultraviolet (UV) and circular dichroism (CD) spectra were recorded at 25 °C using an Applied Photophysics (Leatherhead, Surrey, UK) Chirascan-Plus circular dichroism spectrometer with a 1 mm cuvette. Infrared (IR) spectral data were obtained using a JASCO (Easton, MD, USA) FT/IR-4200 FT-IR spectrometer; 1D and 2D NMR spectra were acquired using Bruker (Billerica, MA, USA) Avance 850 MHz and 800 MHz NMR spectrometers. High-resolution fast atom bombardment MS (HR-FAB-MS) data were measured using a JEOL (Akishima, Tokyo, Japan) JMS-700 HR-MS. The 850 MHz NMR spectrometer and HR-MS were located at the National Center for Interuniversity Research Facilities (NCIRF) in Seoul National University, Republic of Korea.

### 3.2. Bacterial Strains, Plasmids, and Culture Conditions

The bacterial strains and plasmids used in this study are listed in Table S3, and the PCR primers are listed in Table S4. The OMS-producing strain *Streptomyces* sp. SNJ042 was isolated from a sediment sample collected from Jeju Island in Korea [1]. *Escherichia coli* DH5α was used as the host for general cloning, and non-methylating *E. coli* ET12567/pUZ8002 was used for conjugal transfer of plasmids between *E. coli* and *Streptomyces* [26]. Wild-type SNJ042 was maintained on solid YEME medium (4 g of yeast extract, 10 g of malt extract, 4 g of glucose, and 18 g of agar powder per 1 L of sterilized 3.4% seawater) [1], and transformants of SNJ042 strains were selected on ISP4 agar medium supplemented with apramycin (50 μg/mL). All SNJ042 strains were sporulated on ISP4 agar medium at 28 °C. DNA fragments for the plasmid construction were obtained from SNJ042 genomic DNA by PCR using GXL DNA polymerase (Takara, Shiga, Japan) according to the manufacturer’s recommended conditions. The temperature-sensitive *E*. *coli*–*Streptomyces* shuttle vector pKC1139 [27] and the integrative *E*. *coli*–*Streptomyces* shuttle vector pSET152 [27] containing the promoter ermE* were used for gene deletion and expression, respectively.

### 3.3. Draft Genome Sequencing and Bioinformatic Analysis

The genomic DNA of *Streptomyces* sp. SNJ042 was sequenced using an Illumina Hiseq2500 Sequencer and a PacBio RS II Sequencer by Genotech Corp. (Daejeon, Republic of Korea) in combination, and genome assembly was conducted by SMRT analysis (v2.3.0 HGAP.2). Secondary metabolite biosynthetic gene clusters were detected and analyzed using antiSMASH [20]. The NRPS domain and substrate specificities were predicted using the online PKS/NRPS analysis website [28]. The *ohm* cluster was deposited in GenBank under the accession number MN480430.

### 3.4. Construction of Gene Deletion Mutants and Complementation

For in-frame deletion of genes, the recombinant plasmids were constructed by combining the upstream and downstream regions of the target genes as follows: Two DNA fragments, a 1738-bp fragment harboring the upstream region of *ohmL* and a 1677-bp fragment harboring the downstream region of *ohmL*, were amplified with each of the PCR primer pairs delL_LF/delL_LR and delL_RF/delL_RR from SNJ042 genomic DNA. They were ligated into pKC1139 through *Eco*RI–*Xba*I–*Hind*III sites to give the *ohmL* gene deletion plasmid pDel-OhmL. For the deletion of the *ohmK* gene, a 1602-bp *Eco*RI–*Xba*I upstream fragment and a 1435-bp *Xba*I–*Hind*III downstream fragment, amplified using the primer pairs delK_LF/delK_LR and delK_RF/delK_RR, respectively, were cloned into *Eco*RI–*Hind*III-digested pKC1139, generating pDel-OhmK. To create pDel-OhmJ for *ohmJ* gene deletion, a 1630-bp *Mfe*I–*Xba*I downstream fragment and a 1691-bp *Xba*I–*Hind*III upstream fragment obtained separately using the primer pairs delJ_LF/delJ_LR and delJ_RF/delJ_RR were combined into *Eco*RI*Hind*III-digested pKC1139. The recombinant plasmids for gene deletion were transferred by conjugation from non-methylating *E. coli* donor strain ET12567/pUZ8002 to wild-type *Streptomyces* sp. SNJ042. The exconjugants were selected on ISP4 medium supplemented with apramycin (50 μg/mL), and then the desired in-frame deletion mutants via double-crossover homologous recombination were confirmed by PCR amplification (Figure S3).

For complementation experiments, the *ohmK* and *ohmJ* genes were obtained from SNJ042 genomic DNA by PCR using the primer pairs ohmK_F/ohmK_R and ohmJ_F/ohmJ_R, respectively, and each PCR product was digested by the *Bgl*II–*Xba*I site and cloned into a pSET152 integrative vector. The resultant plasmids were designated pOhmK and pOhmJ. The plasmids were conjugated into *ΔohmK* or *ΔohmJ* strains, and apramycin-resistant exconjugants were selected on ISP4 medium.

### 3.5. UPLC-qTOF-HR-MS Analysis of OMS Derivatives

For OMS productions from wild-type, deletion, and complementation SNJ042 strains, spore suspensions were inoculated in liquid A1+C medium (10 g of starch, 4 g of yeast extract, 2 g of peptone, 1 g of CaCO_3_, and 24 g of artificial sea salt per 1 L of sterilized water) in a 250 mL Erlenmeyer flask containing 25 mL of the medium and incubated for 2 days at 30 °C. Next, 1 mL of seed culture was transferred into 25 mL of fresh A1+C medium in a 250 mL Erlenmeyer flask and cultivated at 30 °C with shaking at 200 rpm for 6 days. The liquid culture was extracted with two volumes of ethyl acetate (EtOAc), and the resulting concentrated extract was dissolved in acetonitrile.

The samples were then subjected to UPLC-qTOF-HR-MS analysis, which was performed on a Waters XEVO G2S Q-TOF mass spectrometer coupled with a Waters Acquity I-Class UPLC system equipped with a Peptide BEG C_18_ column (2.1 × 100 mm, 1.7 μm). Gradient elution was applied using solvent A (water with 0.1% trifluoroacetic acid) and solvent B (80% acetonitrile in water with 0.1% trifluoroacetic acid) as the mobile phase, at a flow rate of 0.2 mL/min for 13 min. The MS system was operated in ESI with a positive ionization mode. OMS A and OMS B were eluted.

### 3.6. Large-Scale Culture and Extraction

For preparing dehydroxy-OMS A (**4**), 20 μL of *ΔohmL* strain spore stock was inoculated into 50 mL of A1+C liquid medium and shaken for 200 rpm at 30 °C. After 2 days, 5 mL of the culture was transferred to 150 mL of A1+C liquid media for scale-up. The flask was shaken for 2 days, and a portion of the culture was inoculated into 1 L of amino acid-supplemented A1+C liquid medium (10 g of starch, 4 g of yeast extract, 2 g of peptone, 1 g of CaCO_3_, 1 g of l-Trp, 1 g of l-Phe, 1 g of l-Val, 1 g of l-Thr, 1 g of l-Leu, and 24 g of artificial sea salt per 1 L of sterilized water) and cultivated at 30 °C and 180 rpm. After cultivation for 7 days, the whole culture (30 L) was extracted with EtOAc using a separation funnel. The EtOAc layer was evaporated under low pressure, yielding 10 g of crude extract. The *ΔohmJ* mutant, which produces demethoxy-OMS A (**6**), was cultivated (60 L) and extracted in the same way as described above, yielding 20 g of crude extract.

### 3.7. Purification of OMS Derivatives

The crude extract from the *ΔohmL* mutant was absorbed on Celite and loaded onto 30 g of YMC C_18_ resin. The extract was fractionated by a step gradient solvent system (20%, 40%, 60%, and 100% CH_3_OH in H_2_O; 400 mL for each solvent). Then, a part of each fraction (10 μL) was analyzed by LC–MS using a C_18_ (2) column (Phenomenex Luna, 5 μm, 100 × 4.6 mm). Dehydroxy-OMS A (**4**) was detected in the 100% CH_3_OH fraction. The fraction was then evaporated in vacuo using a rotary evaporator. For purification of **4**, the dried mixture was re-dissolved in 100% CH_3_OH and injected into a semi-preparative HPLC column coupled with a YMC reversed-phase column (Triart, C_18_, 5 μm, 250 × 10 mm). The primary purification was conducted under gradient HPLC conditions (45–65% aqueous CH_3_CN over 40 min with 0.1% trifluoroacetic acid, detection: UV 280 nm, flow rate: 2 mL/min), and a peak containing **4** was eluted at 32.5 min. For further purification, the mixture including **4** was re-injected into a semi-preparative HPLC and purified using a CHIRALPAK column (IC, 5 μm, 250 × 4.6 mm) with gradient HPLC conditions (0–10 min: 30–40% aqueous CH_3_CN, 10–20 min: 50% aqueous CH_3_CN with 0.1% trifluoroacetic acid, detection: UV 280 nm, flow rate: 1 mL/min). Under these conditions, **4** (32.7 mg) was obtained at 15 min. The whole crude extract from the *ΔohmJ* mutant was also fractionated using an identical procedure. The 100% CH_3_OH fraction containing **6** was concentrated under low pressure. The dried fraction was initially subjected to preparative HPLC (Phenomenex Luna, C_18_, 10 μm, 250 × 21.2 mm) under gradient conditions (45–65% aqueous CH_3_CN over 40 min with 0.1% trifluoroacetic acid, detection: UV 280 nm, flow rate: 10 mL/min). A peak including **6** was collected at 26 min and re-injected into a semi-preparative HPLC for further purification. By using a reversed-phase C_18_ (2) HPLC column (Kromasil, 5 μm, 250 × 10 mm) with gradient HPLC conditions (0–20 min: 40–56% aqueous CH_3_CN, 20–40 min: 60% aqueous CH_3_CN with 0.1% trifluoroacetic acid, detection: UV 280 nm, flow rate: 2 mL/min), pure demethoxy-OMS A (**6**) (33.0 mg) was acquired at 31 min after injection.

### 3.8. Characterization of OMS Derivatives

Dehydroxy-ohmyungsamycin A (**4**): yellow powder, ${\left[\alpha \right]}_{D}^{20}$ −29.3 (c 0.1, CH_3_OH); UV (CH_3_OH) λ_max_ (log *ε*) 205 (3.18), 207 (3.17), 286 (2.39) nm; ECD (c 0.1 × 10^−4^ M, CH_3_OH) λ_max_ (Δ*ε*) 232 (−1.55) nm; IR (neat) ν_max_ 3702, 3671, 3349, 3300, 2971, 2869, 1655, 1055, 1011 cm^−1^; for ^1^H and ^13^C NMR spectral data, see Table 1; HR-FAB-MS [M+H]^+^ *m*/*z* at 1442.9027 (calcd for C_75_H_120_N_13_O_15_, 1442.9027).

Demethoxy-ohmyungsamycin A (**6**): yellow powder, ${\left[\alpha \right]}_{D}^{20}$ -26.1 (c 0.1, CH_3_OH); UV (CH_3_OH) λ_max_ (log *ε*) 205 (3.18), 207 (3.18), 283 (2.40) nm; ECD (c 0.1 × 10^−4^ M, CH_3_OH) λ_max_ (Δ*ε*) 231 (−1.87) nm; IR (neat) ν_max_ 3702, 3672, 3357, 3290, 2971, 2869, 1661, 1055, 1011 cm^−1^; for ^1^H and ^13^C NMR spectral data, see Table 2; HR-FAB-MS [M+H]^+^ *m*/*z* at 1428.8860 (calcd for C_74_H_118_N_13_O_15_, 1428.8870).

### 3.9. Minimum Inhibitory Concentration (MIC) Determination by REMA

The MICs of the compounds were determined as described previously with modifications [29]. Briefly, *M*. *tuberculosis* mc^2^ 6230 strain was grown at 37 °C in Middlebrook 7H9 broth containing 0.2% casamino acids and 0.24 μg/mL pantothenate as supplements. A 100 μL aliquot of media was added to every well of a 96-well microtiter plate, and two-fold serial dilutions of the antibiotics were added directly to each well. Then, the plates were incubated at 37 °C for 5 days. Resazurin (0.025% [*wt*/*vol*]) was added to each well, and the fluorescence was measured (ex/em 560/590 nm) using a SpectraMax^®^ M3 Multi-Mode Microplate Reader (Molecular Devices, CA, USA). The MIC values were calculated using Prism 6 (GraphPad Software, Inc., La Jolla, CA).

### 3.10. Sulforhodamine B (SRB) Assay for the Determination of Proliferation Inhibition of Cancer Cells

Cells were seeded in 96-well plates, incubated for 24 h, and then fixed (for zero-day controls) or treated with the test compounds for 72 h. After incubation, the cells were fixed with 10% trichloroacetic acid (TCA), dried, and stained in 0.4% SRB in 1% acetic acid solution [30]. Unbound dye was washed, and the stained cells were dried and suspended in 10 mM Tris (pH 10.0). The absorbance was measured at 515 nm, and the cell proliferation was determined as follows: cell proliferation (%) = (average absorbance _compound_ − average absorbance _zero-day_)/(average absorbance _control_ − average absorbance _zero-day_) × 100. The IC_50_ values were calculated by non-linear regression analysis using TableCurve 2D v5.01 software (Systant Software Inc., Richmond, CA, USA).

## 4. Conclusions

The biosynthetic gene cluster for OMSs, whose products show prominent antituberculosis and antiproliferative activities against *M*. *tuberculosis* and human cancer cell lines, was revealed through draft genome sequencing of their producer strain *Streptomyces* sp. SNJ042. In addition, OhmL (P450), OhmK (TDO), and OhmJ (*O*-MT) were selected as candidates for forming the non-proteinogenic amino acids *β*-hydroxy-l-Phe and 4-methoxy-l-Trp in OMSs. To identify the role of the enzyme candidates, the genes were inactivated via in-frame deletion, and the accumulated products were analyzed by UPLC-qTOF-HR-MS, showing that OhmL, OhmK, and OhmJ are an l-Phe *β*-hydroxylase, an l-Trp 4-hydroxylase, and an l-Trp 4-*O*-MT, respectively. On the basis of a spectroscopic analysis, the chemical structures of the OMS derivatives, dehydroxy-OMS A (**4**) and demethoxy-OMS A (**6**), were elucidated, and their antituberculosis and antiproliferative activities were also measured. Particularly, dehydroxy-OMS A (**4**) showed stronger growth inhibition activity against *M*. *tuberculosis*, whereas its cytotoxicity was decreased compared to that of wild-type OMS A (**1**). These results indicate that the presence of a hydroxy group at the *β*-carbon of Phe affects the bioactivity of OMSs.

## 5. Patents

Provisional patent applications covering this work have been filed.

## Figures and Tables

**Table 1:** ^1^H and ^13^C NMR data for **4** in pyridine-*d*_5_.

	Position	*δ*_C_*^a^*, Type	*δ*_H_*^b^*, Mult (*J* in Hz)		Position	*δ*_C_*^a^*, Type	*δ*_H_*^b^*, Mult (*J* in Hz)
Val-1	1	174.6, C		Val-6	38	173.0, C	
	2	58.1, CH	4.72, dd (9.0, 7.5)		39	54.4, CH	4.96, dd (9.5, 9.5)
	2-NH		9.17, d (9.0)		39-NH		9.46, d (9.5)
	3	33.0, CH	2.22, m		40	31.8, CH	2.42, m
	4	18.9, CH_3_	1.13, m*^c^*		41	19.7, CH_3_	1.14, d (7.0)
	5	18.8, CH_3_	0.99, d (7.0)		42	18.9, CH_3_	1.03, d (7.0)
Phe-2	6	172.8, C		*N*-Me-Leu-7	43	171.7, C	
	7	56.8, CH	5.49, ddd (9.5, 8.0, 4.5)		44	54.7, CH	5.68, dd (7.5, 7.5)
	7-NH		10.40, d (8.0)		45a	39.0, CH_2_	1.75, m
	8a	38.2, CH_2_	3.80, dd (14.0,4.0)		45b		1.58, m
	8b		3.28, dd (14.0, 9.5)		46	25.1, CH	1.38, m
	9	139.3, C			47	23.1, CH_3_	0.69, d (6.5)
	10	129.8, CH	7.34, d (7.5)		48	21.9, CH_3_	0.66, d (6.5)
	11	128.9, CH	7.40, dd (7.5, 7.0)		49	31.3, CH_3_	3.53, s
	12	126.7, CH	7.24, dd (7.0, 7.0)	Val-8	50	173.6, C	
	13	128.9, CH	7.40, dd (7.5, 7.0)		51	55.7, CH	5.28, dd (8.5, 8.5)
	14	129.8, CH	7.34, d (7.5)		51-NH		8.08, d (8.5)
Val-3	15	174.2, C			52	31.1, CH	2.58, m
	16	58.6, CH	5.15, dd (9.5, 9.5)		53	19.9, CH_3_	1.16, d (7.0)
	16-NH		8.04, d (9.5)		54	19.2, CH_3_	1.20, d (6.5)
	17	32.8, CH	2.66, m	*N*-Me-Thr-9	55	170.6, C	
	18	20.2, CH_3_	1.11, d (7.0)		56	62.4, CH	5.57, d (3.5)
	19	19.9, CH_3_	1.31, d (7.0)		57	66.6, CH	5.07, qd (6.5, 3.5)
*N*-Me-4-OMe-Trp-4	20	169.8, C			58	20.5, CH_3_	1.35, d (6.5)
	21	70.7, CH	4.60, dd (11.0, 4.5)		59	34.8, CH_3_	3.71, s
	22a	27.2, CH_2_	4.45, dd (13.5, 4.5)	Thr-10	60	171.3, C	
	22b		4.33, dd (13.5, 11.0)		61	52.6, CH	5.94, dd (9.0, 2.5)
	23	112.9, C			61-NH		10.31, d (9.0)
	24	124.3, CH	7.19, d (1.5)		62	69.4, CH	6.10, qd (6.5, 2.5)
	24-NH		11.80, d (1.5)		63	16.7, CH_3_	1.56, d (6.5)
	25	139.6, C		Val-11	64	172.6, C	
	26	105.9, CH	7.30, d (7.5)		65	59.1, CH	5.29, dd (8.5, 8.5)
	27	122.8, CH	7.27, dd (7.5, 7.5)		65-NH		9.92, d (8.5)
	28	99.6, CH	6.68, d (7.5)		66	31.5, CH	2.27, m
	29	154.9, C			67	19.5, CH_3_	0.94, d (7.0)
	29-OMe	55.5, CH_3_	3.85, s		68	18.8, CH_3_	0.93, d (6.5)
	30	118.5, C		*N*-Me-Val-12	69	168.1, C	
	31	40.9, CH_3_	2.51, s		70	67.9, CH	4.33, d (6.0)
*N*-Me-Val-5	32	169.5, C			71	30.9, CH	2.64, m
	33	71.0, CH	3.21, d (8.5)		72	19.3, CH_3_	1.25, d (7.0)
	34	29.0, CH	3.03, m		73	18.5, CH_3_	1.19, d (7.0)
	35	21.9, CH_3_	1.22, d (6.5)		74	32.4, CH_3_	2.99, s
	36	19.7, CH_3_	1.02, d (6.5)				
	37	40.0, CH_3_	3.20, s				

*^a^* 800 MHz, *^b^* 200 MHz, *^c^* overlapped.

**Table 2:** ^1^H and ^13^C NMR data for **6** in pyridine-*d*_5_.

	Position	*δ*_C_*^a^*, Type	*δ*_H_*^b^*, Mult (*J* in Hz)		Position	*δ*_C_*^a^*, Type	*δ*_H_*^b^*, Mult (*J* in Hz)
Val-1	1	174.3, C		Val-6	38	173.1, C	
	2	58.4, CH	4.65, dd (8.5, 8.0)		39	54.4, CH	4.96, dd (9.0, 8.5)
	2-NH		9.26, d (8.5)		39-NH		9.36, d (8.5)
	3	33.0, CH	2.22, m		40	32.0, CH	2.39, m*^c^*
	4	19.0, CH_3_	0.92, m*^c^*		41	19.3, CH_3_	1.12, m*^c^*
	5	18.9, CH_3_	1.12, m*^c^*		42	19.3, CH_3_	1.02, m*^c^*
*β*-OH-Phe-2	6	172.7, C		*N*-Me-Leu-7	43	171.6, C	
	7	60.1, CH	5.50, m*^c^*		44	54.8, CH	5.61, m*^c^*
	7-NH		9.73, br s		45a	38.9, CH_2_	1.73, m*^c^*
	8	73.3, CH	5.92, m*^c^*		45b		1.50, m*^c^*
	9	143.0, C			46	25.1, CH	1.36, m*^c^*
	10	127.0, CH	7.65, m*^c^*		47	23.3, CH_3_	0.63, d (6.0)
	11	128.6, CH	7.38, m*^c^*		48	22.0, CH_3_	0.59, d (6.0)
	12	127.6, CH	7.24, m*^c^*		49	31.4, CH_3_	3.49, s
	13	128.6, CH	7.38, m*^c^*	Val-8	50	173.5, C	
	14	127.0, CH	7.65, m*^c^*		51	55.7, CH	5.27, dd (8.5, 8.0)
Val-3	15	174.4, C			51-NH		8.08, d (8.5)
	16	58.5, CH	5.43, dd (9.5, 9.0)		52	31.2, CH	2.55, m*^c^*
	16-NH		8.15, d (9.5)		53	19.3, CH_3_	1.19, m*^c^*
	17	33.1, CH	2.68, m		54	19.3, CH_3_	1.14, m*^c^*
	18	20.2, CH_3_	1.22, m*^c^*	*N*-Me-Thr-9	55	170.6, C	
	19	19.8, CH_3_	1.35, d (6.0)		56	62.5, CH	5.60, d (3.5)
*N*-Me-Trp-4	20	169.4, C			57	66.5, CH	5.06, m
	21	70.7, CH	4.21, d (10.5, 3.0)		58	20.5, CH_3_	1.32, d (6.0)
	22a	25.1, CH_2_	4.40, m*^c^*		59	34.7, CH_3_	3.71, s
	22b		4.30, dd (15.0, 3.0)	Thr-10	60	171.4, C	
	23	113.1, C			61	52.6, CH	5.92, m
	24	124.5, CH	7.35, m*^c^*		61-NH		10.29, d (8.5)
	24-NH		11.87, br s		62	69.8, CH	6.09, m
	25	137.8, C			63	16.8, CH_3_	1.56, d (6.5)
	26	127.0, CH	7.65, d (7.5)	Val-11	64	172.6, C	
	27	128.7, CH	7.38, m*^c^*		65	59.2, CH	5.23, m
	28	119.0, CH	7.24, m*^c^*		65-NH		9.96, d (8.5)
	29	119.4, CH	7.86, d (7.5)		66	31.4, CH	2.25, m
	30	128.5, C			67	19.0, CH_3_	0.92, m*^c^*
	31	41.0, CH_3_	2.46, s		68	19.0, CH_3_	0.92, m*^c^*
*N*-Me-Val-5	32	169.5, C		*N*-Me-Val-12	69	168.0, C	
	33	71.1, CH	3.28, d (7.0)		70	67.8, CH	4.37, m*^c^*
	34	29.3, CH	3.06, m		71	30.9, CH	2.65, m*^c^*
	35	22.0, CH_3_	1.25, m*^c^*		72	19.3, CH_3_	
	36	19.7, CH_3_	1.05, m*^c^*		73	18.5, CH_3_	1.20, m*^c^*
	37	40.0, CH_3_	3.19, s		74	32.3, CH_3_	2.99, s

*^a^* 850 MHz, *^b^* 212.5 MHz, *^c^* overlapped.

**Table 3:** Antituberculosis and antiproliferative activities against *M. tuberculosis* and human cancer cell lines of OMS A (**1**), dehydroxy-OMS A (**4**), and demethoxy-OMS A (**6**). IC_50_: half maximal inhibitory concentration, MIC_50_: the lowest concentration of antibiotics at which 50% of the isolates were inhibited.

	MIC_50_ (nM)	IC_50_ (μM)					
Cell Lines/Strain	*Mycobacterium Tuberculosis* mc^2^ 6230	A549	HCT 116	MDA-MB-231	SK-HEP-1	SNU 638	MRC-5
OMS A (**1**)	29.3	11.26	10.91	14.90	14.41	13.27	>50
Dehydroxy-OMS A (**4**)	4.9	29.87	25.10	27.26	33.26	29.38	>50
Demethoxy-OMS A (**6**)	40.2	9.56	7.68	9.52	10.11	11.58	>50
Etoposide	-	0.42	1.23	7.79	1.11	1.24	14.68
Isoniazid (INH)	142.9	-	-	-	-	-	-
Ethambutol (EMB)	2700	-	-	-	-	-	-

## Supplementary Materials

The supplementary materials are available online at https://www.mdpi.com/2218-273X/9/11/672/s1.
