# Supplementary material for: Characterization of the Ohmyungsamycin Biosynthetic Pathway and Generation of Derivatives with Improved Antituberculosis Activity
Source: Biomolecules. 2019 Oct 30;9(11):672. doi: 10.3390/biom9110672 (PMC6920865; doi:10.3390/biom9110672)
Supplement: Supplementary file 1 [file biomolecules-09-00672-s001.pdf]

Supplementary Materials

Characterization of the ohmyungsamycin biosynthetic pathway and generation of the derivatives with improved antituberculosis activity

Eunji Kim [1,†], Yern-Hyerk Shin [2,†], Tae Ho Kim [3], Woong Sub Byun [2], Jinsheng Cui [2], Young Eun Du [2], Hyung-Ju Lim [2], Myoung Chong Song [1], An Sung Kwon [4], Sang Hyeon Kang [4], Jongheon Shin [2], Sang Kook Lee [2], Jichan Jang [3], Dong-Chan Oh [2,*] and Yeo Joon Yoon [1,*]

[1] Department of Chemistry and Nanoscience, Ewha Womans University, Seoul 03760, Republic of Korea; ejkim0618@ewha.ac.kr (E.K.); smch517@ewha.ac.kr (M.C.S.)

[2] Natural Products Research Institute, College of Pharmacy, Seoul National University, Seoul 08826, Republic of Korea; itsue00@snu.ac.kr (Y.-H.S.); sky_magic@naver.com (W.S.B.); cuijs@snu.ac.kr (J.C.); dye0302@snu.ac.kr (Y.E.D.); limju012@snu.ac.kr (H.-J.L.); shinj@snu.ac.kr (J.S.); sklee61@snu.ac.kr (S.K.L.)

[3] Division of Applied Life Science (BK21plus Program), Gyeongsang National University, Jinju 52828, Republic of Korea; taeho12349@gmail.com (T.H.K.); jichanjang@gnu.ac.kr (J.J.)

[4] iNtRON Biotechnology, Inc., Seongnam-si, Gyeonggi-do 13202, Republic of Korea; kwon4053@intron.co.kr (A.S.K.); kangsh0403@naver.com (S.H.K)

* Correspondence: dongchanoh@snu.ac.kr; Tel.: +82-2-880-2491 (D.-C.O.)
joonyoon@ewha.ac.kr; Tel.: +82-2-3277-4082 (Y.J.Y.)

† These authors contributed equally.

Table of Contents

S2. Table of Contents
S3. Figure S1. Conserved sequence regions from alignment comparisons of P450s implicated in the β-hydroxylation of PCP-bound amino acid residues.
S4. Figure S2A. Structures of marine-derived cyclic peptides containing 5-hydroxy-L-Trp moiety.
Figure S2B. Structures of compounds containing L-Trp derivatives.
S5. Figure S3. Schematic representation of mutant strain construction by in-frame deletion.
S6. Figure S4A. Structural assignment of OMS A (**1**) and OMS B (**2**) produced from SNJ042 wild type.
S7. Figure S4B. Structural assignment of dehydroxylated OMS derivatives **4** and **5** produced from *ohmL* deletion mutant strain.
S8. Figure S4C. Structural assignment of demethoxylated OMS derivatives **6** and **7** produced from *ohmK* deletion mutant strain.
S9. Figure S4D. Structural assignment of demethoxylated OMS derivatives **6** and **7** produced from *ohmJ* deletion mutant strain.
S10. Figure S5. HR-FAB-MS data of **4**.
S11. Figure S6. ^{1}H NMR spectrum data of **4** at 800 MHz in pyridine-d_5.
S12. Figure S7. ^{13}C NMR spectrum data of **4** at 200 MHz in pyridine-d_5.
S13. Figure S8. COSY NMR spectrum data of **4** at 800 MHz in pyridine-d_5.
Figure S9. TOCSY NMR spectrum data of **4** at 800 MHz in pyridine-d_5.
S14. Figure S10. HSQC NMR spectrum data of **4** at 800 MHz in pyridine-d_5.
Figure S11. HMBC NMR spectrum data of **4** at 800 MHz in pyridine-d_5.
S15. Figure S12. ROESY NMR spectrum data of **4** at 800 MHz in pyridine-d_5.
S16. Figure S13. Comparing CD spectra data of **1**, **4**, and **6**.
S17. Figure S14. Phylogenetic relationships of known TDOs from secondary metabolite biosynthesis, including OhmK.
S18. Figure S15. HR-FAB-MS data of **6**.
S19. Figure S16. ^{1}H NMR spectrum data of **6** at 850 MHz in pyridine-d_5.
S20. Figure S17. ^{13}C NMR spectrum data of **6** at 212.5 MHz in pyridine-d_5.
S21. Figure S18. COSY NMR spectrum data of **6** at 850 MHz in pyridine-d_5.
Figure S19. TOCSY NMR spectrum data of **6** at 850 MHz in pyridine-d_5.
S22. Figure S20. HSQC NMR spectrum data of **6** at 850 MHz in pyridine-d_5.
Figure S21. HMBC NMR spectrum data of **6** at 850 MHz in pyridine-d_5.
S23. Figure S22. ROESY NMR spectrum data of **6** at 850 MHz in pyridine-d_5.
S24. Table S1. Deduced function of ORFs in the ohmyungsamycin biosynthetic gene cluster
S25. Table S2. Substrate specificity sequences for adenylation (A) domains from ohmyungsamycin NRPS
S26. Table S3. Bacterial strains and plasmids used in this study
S27. Table S4. Primers used in this study
S28. Table S5. ^{1}H NMR data for minor conformer of **4** in pyridine-d_5
S29. Figure S23. Key ROESY correlations of (A) major and (B) minor conformers of dehydroxy-OMS A (**4**).
S30. Table S6. ^{1}H NMR data for minor conformer of **6** in pyridine-d_5
S31. Figure S24. Key ROESY correlations of (A) major and (B) minor conformers of demethoxy-OMS A (**6**).
S32. References

Figure S1. Conserved sequence regions from alignment comparisons of P450s implicated in the β-hydroxylation of PCP-bound amino acid residues. Numbering is indicated for OhmL. Identity residues are shown as bold and blue. Similar residues and exceptions are shown in normal and italics, respectively.

P450s	B-B$_2$ loop N-term.		B-B$_2$ loop C-term.						F-helix				G-helix					I-helix			β-1 sheet		
	69	72	82	83	84	86	89	90	169	170	171	172	185	186	187	188	191	230	231	237	284	285	286
OhmL	**G**	L	A	A	**G**	**M**	V	T	T	**A**	F	G	**A**	H	*V*	*D*	S	**N**	**C**	**G**	A	M	**H**
Ecu1	**G**	L	A	A	*D*	**M**	V	T	V	**A**	F	G	**A**	H	*V*	**E**	S	**N**	**C**	**G**	A	M	**H**
Sky32	**G**	L	A	A	**G**	**M**	V	T	S	**A**	L	S	**A**	R	N	**E**	L	**N**	**C**	**G**	A	M	**H**
OxyD	**G**	I	S	G	**G**	**M**	V	S	H	**A**	F	G	**A**	H	T	**E**	V	**N**	**C**	**G**	A	M	**H**
NovI	**G**	L	A	S	**G**	**M**	V	T	H	**A**	W	S	**A**	K	N	**E**	L	**N**	**C**	**G**	S	L	**H**
NikQ	**G**	L	A	A	**G**	**M**	I	T	F	**A**	W	S	**A**	H	T	**E**	L	**N**	**C**	**G**	V	M	**H**
Ecm12	**G**	L	A	G	**G**	**M**	V	T	L	**A**	L	S	**A**	R	N	**E**	G	**N**	**C**	**G**	A	M	**H**
Consensus	**G**	IL	SG<br>A	SG<br>A	**G**	**M**	VI<br>L	ST	H	**A**	W<br>FL	SG<br>A	**A**	KR<br>H	NT<br>S	**E**	LV<br>SG	**N**	**C**	**G**	SA<br>V	LM	**H**

Accession numbers: Ecu1 (AIW58896.1, *Nonomuraea* sp. MJM5123), Sky32 (AEA30275.1, *Streptomyces* sp. Acta 2897), OxyD (CAC48370.1, *Amycolatopsis balhimycina* DSM 5908), NovI (AAF67502.1, *Streptomyces niveus* NCIMB 9219), NikQ (CAB75339.1, *Streptomyces tendae* Tue901), Ecm12 (BAE98161.1, *Streptomyces lasaliensis*)

Figure S2A. Structures of marine-derived cyclic peptides containing 5-hydroxy-L-Trp moiety. Hydroxy groups at position 5 on the indole are marked in blue.

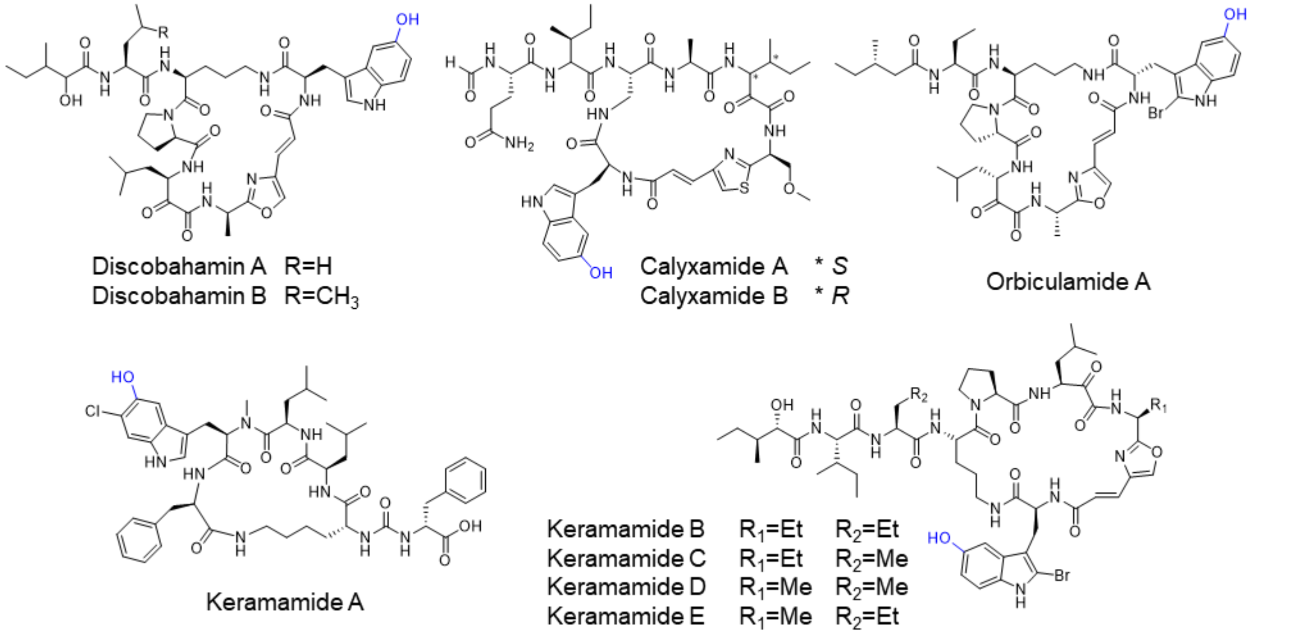

Figure S2B. Structures of compounds containing L-Trp derivatives which are generated by Trp 2,3-dioxygenase catalysis via the kynurenine pathway. Disguised Trp moieties are indicated in red.

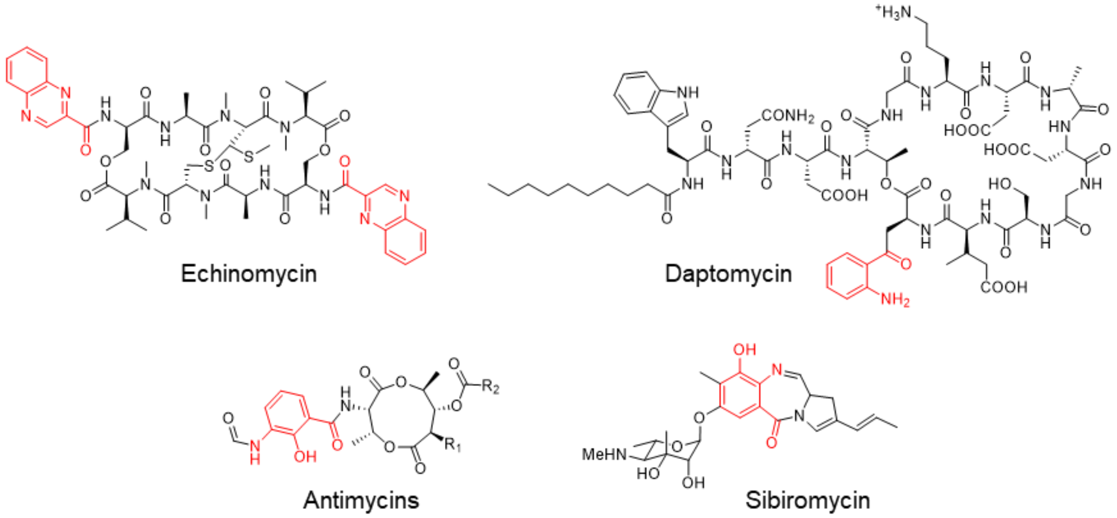

Figure S3. Schematic representations of mutant strain construction by in-frame deletion.

Construction of modification gene deletion mutant strains *ΔohmL*(i), *ΔohmK* (iii), and *ΔohmJ* (v), respectively, and verification of each mutant strain by PCR analysis (ii), (iv), and (vi).

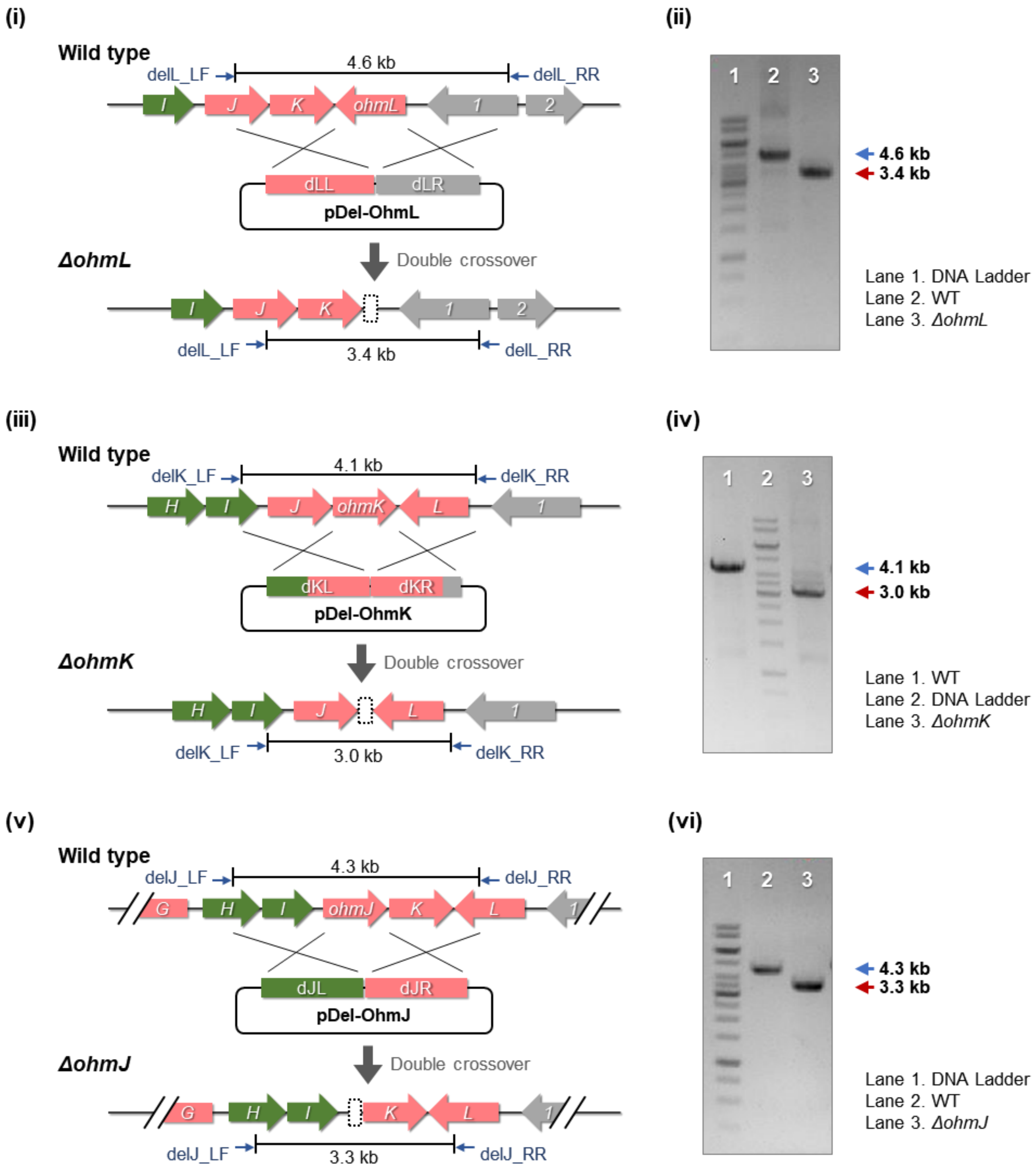

Figure S4A. Structural assignment of OMS A (**1**) and OMS B (**2**) produced from SNJ042 wild type.

(i) MS/MS fragmentation pattern and MS/MS spectra of OMS A (**1**) from wild type strain.
(ii) MS/MS fragmentation pattern and MS/MS spectra of OMS B (**2**) from wild type strain.

(i)

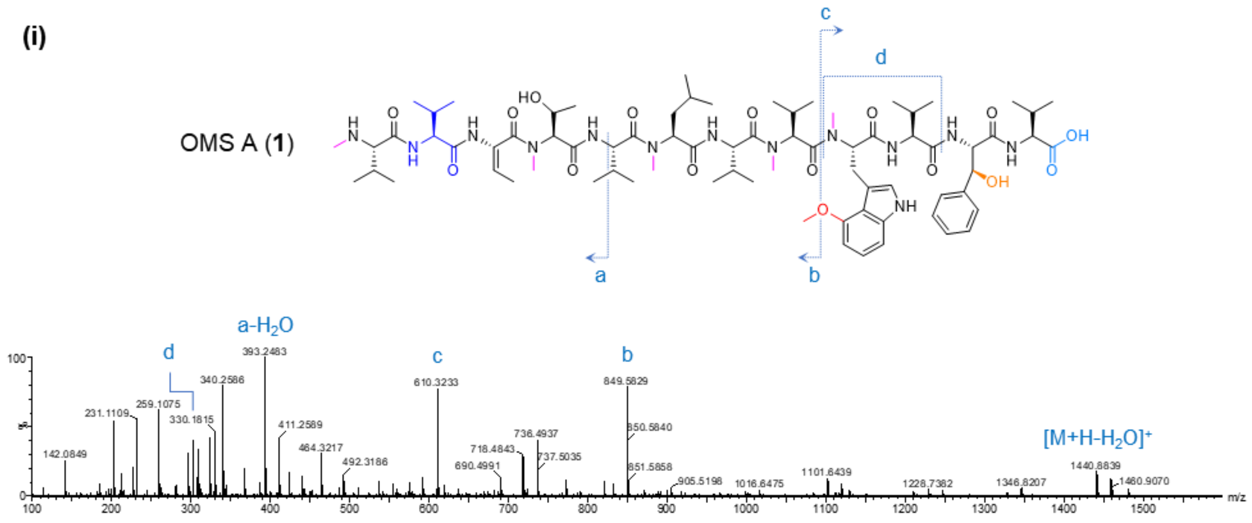

(ii)

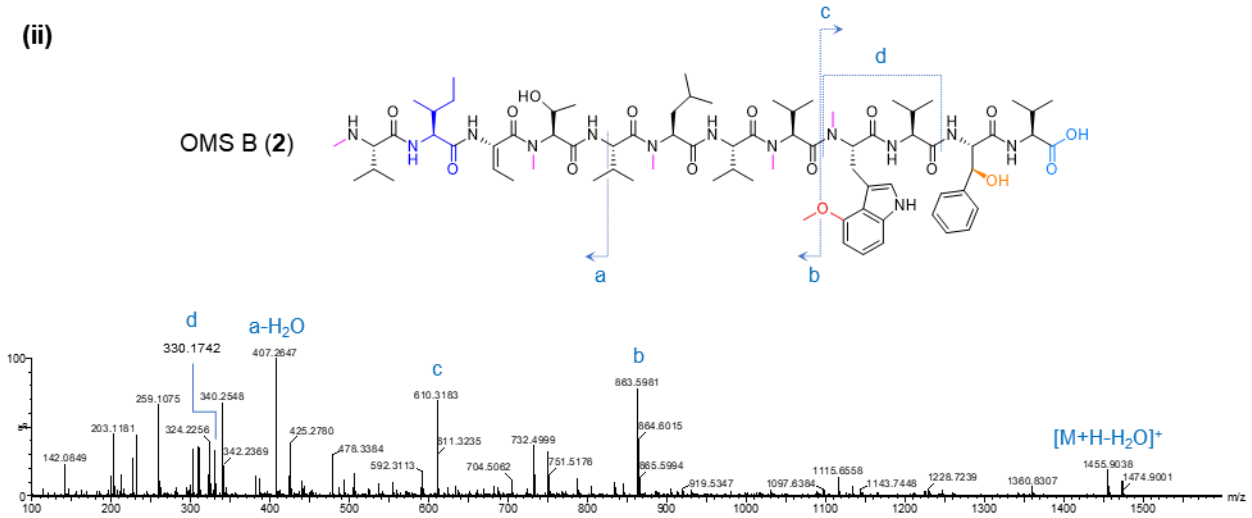

Figure S4B. Structural assignment of dehydroxylated OMS derivatives **4** and **5** produced from *ohmL* deletion mutant strain.

(i) MS/MS fragmentation pattern and MS/MS spectra of dehydroxy-OMS A (**4**) from *ΔohmL* strain.

(ii) MS/MS fragmentation pattern and MS/MS spectra of dehydroxy-OMS B (**5**) from *ΔohmL* strain.

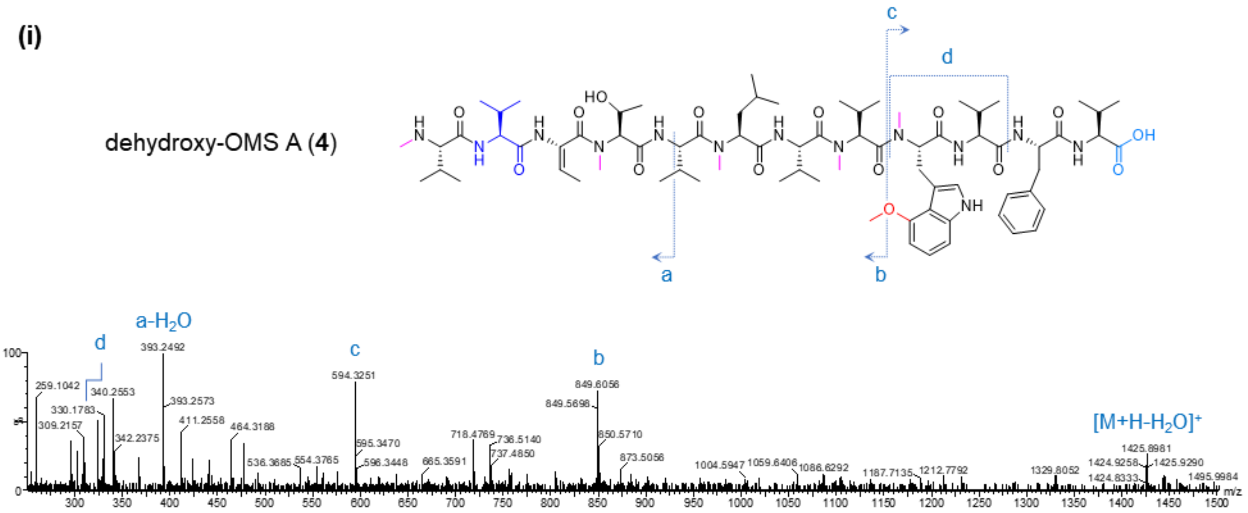

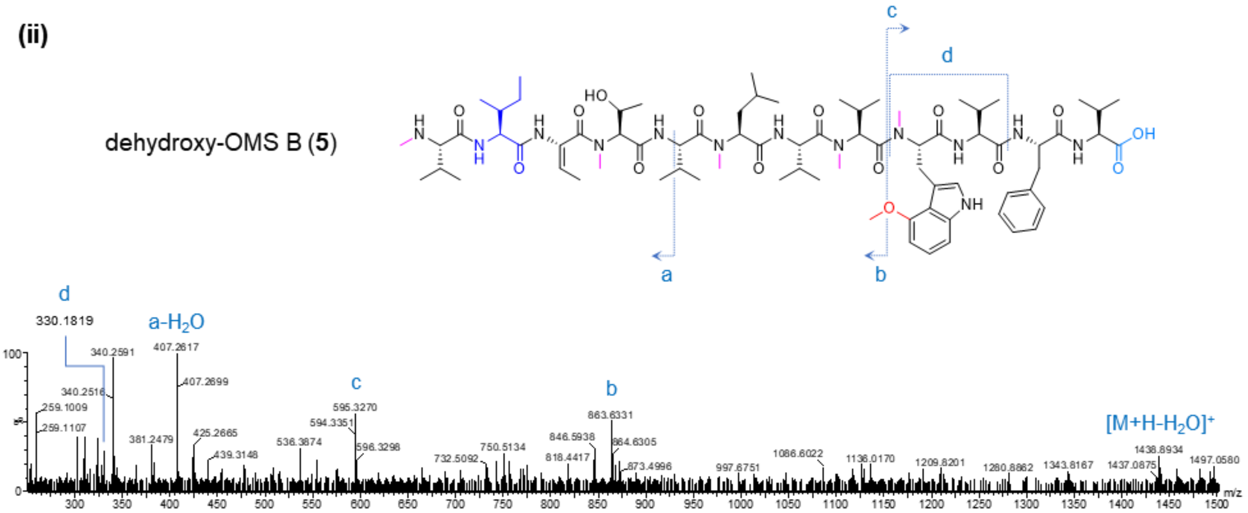

Figure S4C. Structural assignment of demethoxylated OMS derivatives **6** and **7** produced from *ohmK* deletion mutant strain.

(i) MS/MS fragmentation pattern and MS/MS spectra of demethoxy-OMS A (**6**) from *ΔohmK* strain.

(ii) MS/MS fragmentation pattern and MS/MS spectra of demethoxy-OMS B (**7**) from *ΔohmK* strain.

(i)

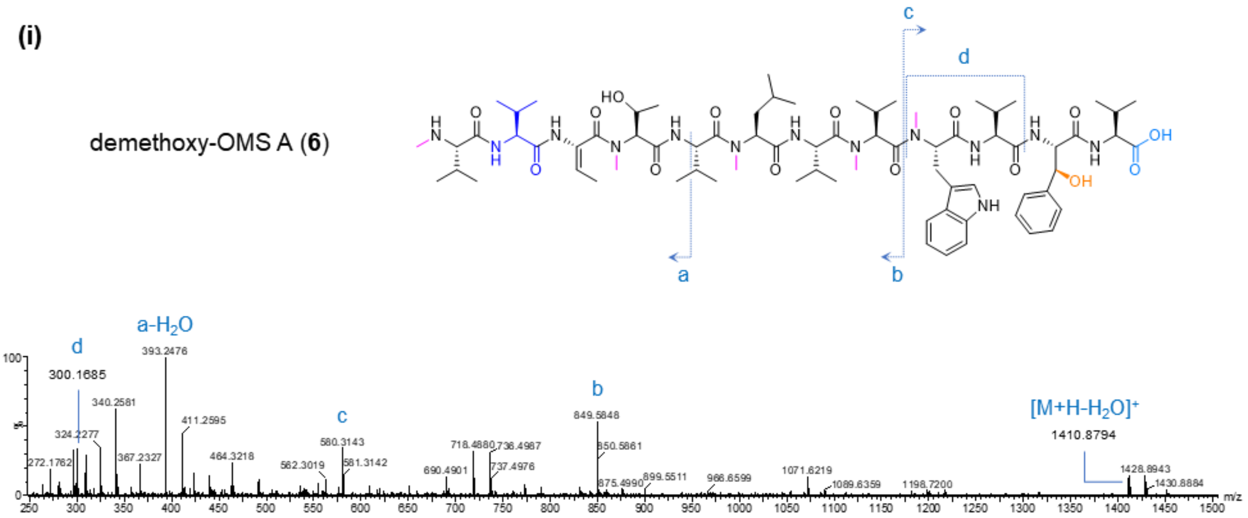

(ii)

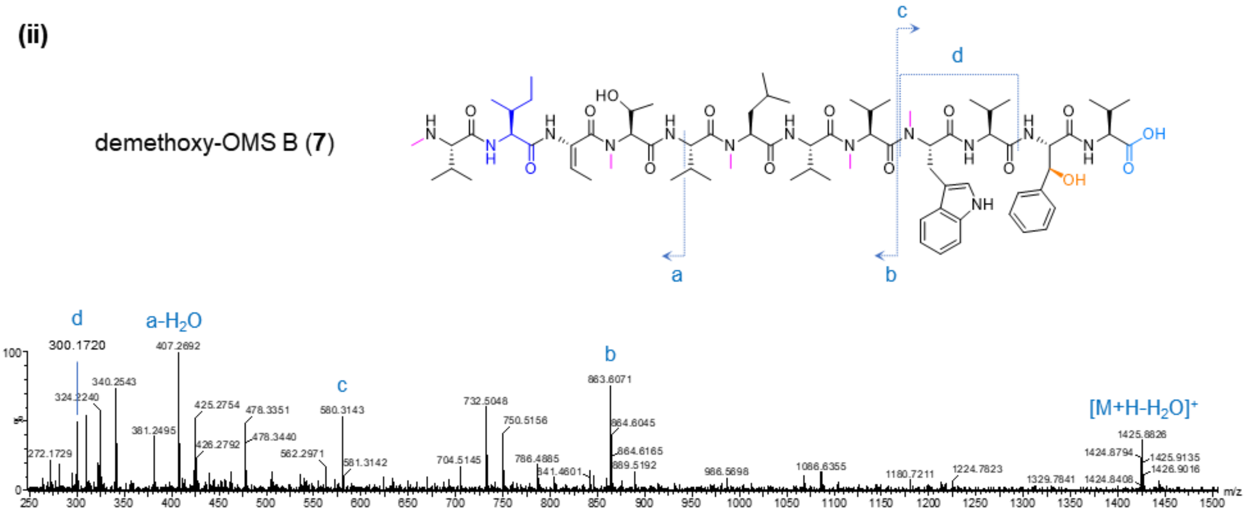

Figure S4D. Structural assignment of demethoxylated OMS derivatives **6** and **7** produced from *ohmJ* deletion mutant strain.

(i) MS/MS fragmentation pattern and MS/MS spectra of demethoxy-OMS A (**6**) from *ΔohmJ* strain.
(ii) MS/MS fragmentation pattern and MS/MS spectra of demethoxy-OMS B (**7**) from *ΔohmJ* strain.

(i)

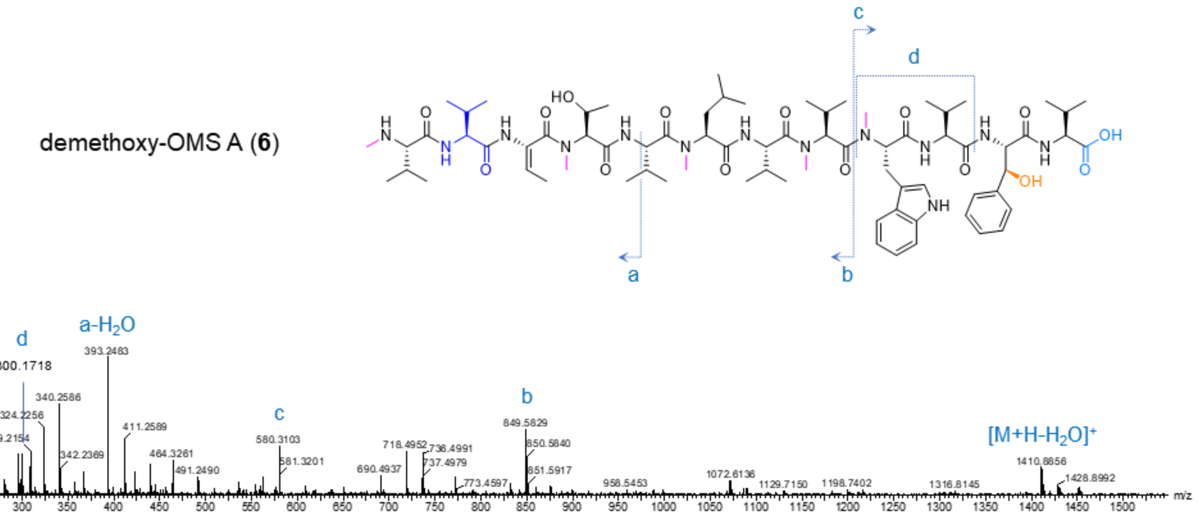

(ii)

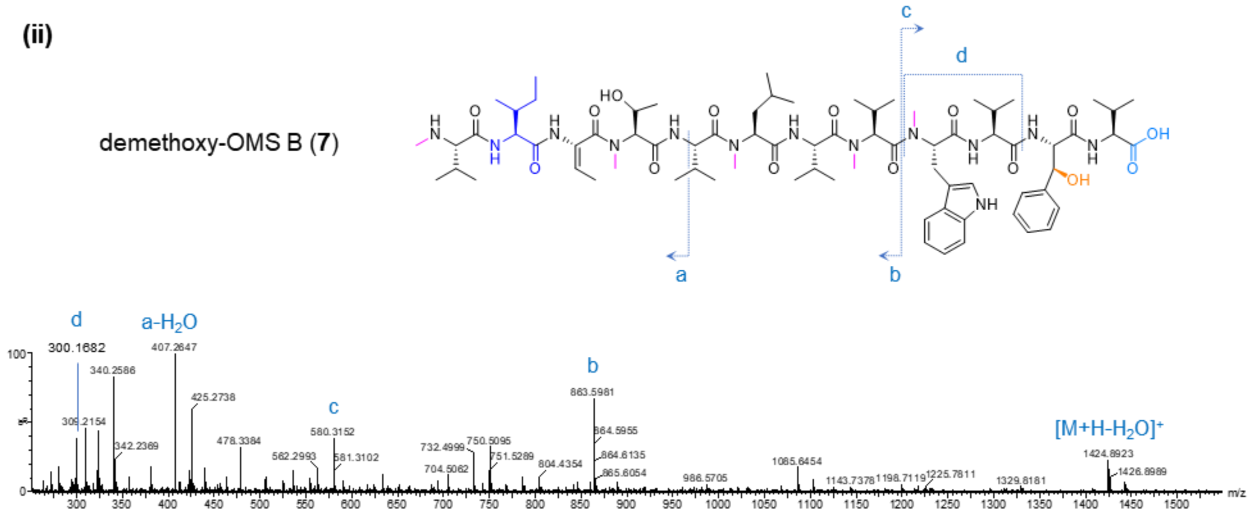

Figure S5. HR-FAB-MS data of **4**.

```
[ Elemental Composition ]                                                    Page: 1
Data   : FAB-S236                    Date : 15-Mar-2019 21:35
Sample: 865.1441
Note : m-NBA
Inlet : Direct                       Ion Mode : FAB+
RT : 1.80 min                        Scan#: (19,149)
Elements : C 100/0, H 200/0, N 15/10, O 20/10
Mass Tolerance      : 20ppm, 5mmu if m/z < 250, 10mmu if m/z > 500
Unsaturation (U.S.) : -0.5 - 50.0

Observed m/z Int%    Err[ppm / mmu]     U.S.   Composition
 1442.9027   100.0       -4.6 /  -6.7   31.0   C 83 H 118 N 12 O 10
                         +4.1 /  +5.9   31.5   C 82 H 116 N 13 O 10
                         +3.2 /  +4.6   31.0   C 84 H 118 N 10 O 11
                         -1.8 /  -2.7   27.0   C 78 H 118 N 14 O 12
                         +6.9 /  +9.9   27.5   C 77 H 116 N 15 O 12
                         -2.8 /  -4.0   26.5   C 80 H 120 N 11 O 13
                         +5.9 /  +8.6   27.0   C 79 H 118 N 12 O 13
                         +0.0 /  +0.0   22.5   C 75 H 120 N 13 O 15
                         -0.9 /  -1.3   22.0   C 77 H 122 N 10 O 16
                         -5.9 /  -8.5   18.0   C 71 H 122 N 14 O 17
                         +2.8 /  +4.0   18.5   C 70 H 120 N 15 O 17
                         -6.8 /  -9.9   17.5   C 73 H 124 N 11 O 18
                         +1.9 /  +2.7   18.0   C 72 H 122 N 12 O 18
                         -4.1 /  -5.8   13.5   C 68 H 124 N 13 O 20
                         +4.7 /  +6.7   14.0   C 67 H 122 N 14 O 20
```

Figure S6. ^{1}H NMR spectrum data of **4** at 800 MHz in pyridine-d_5.

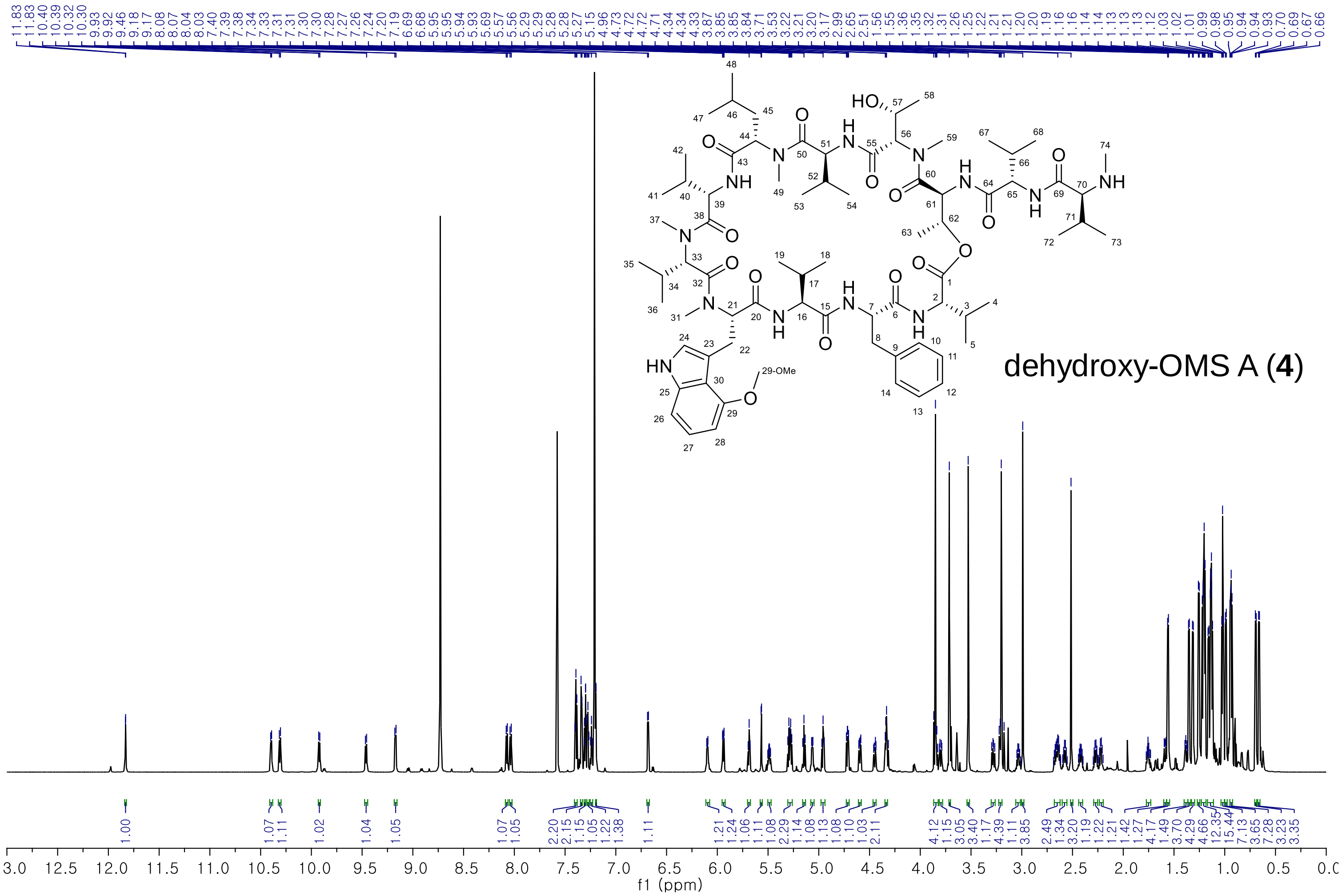

Figure S7. ^{13}C NMR spectrum data of **4** at 200 MHz in pyridine-d_5.

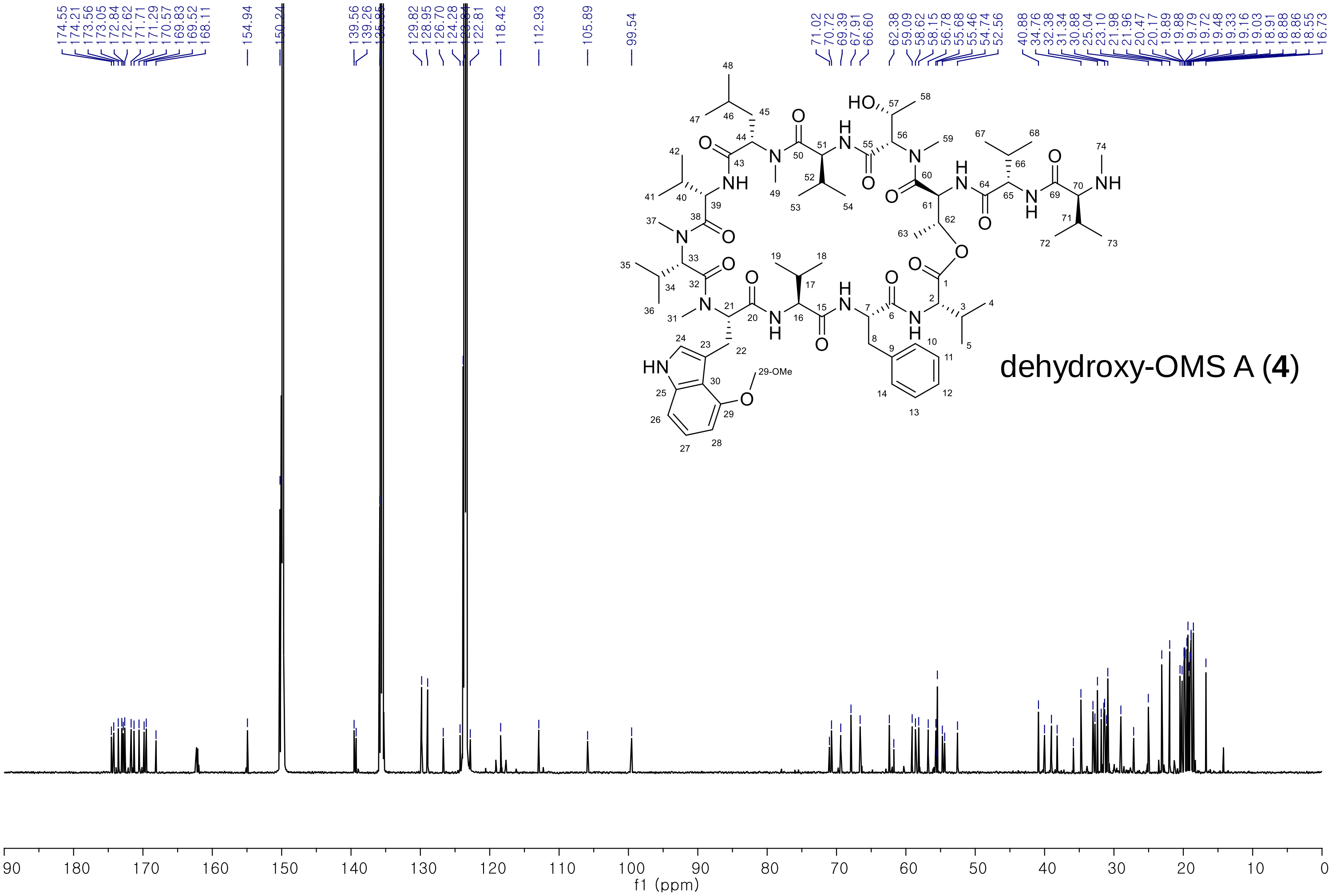

Figure S8. COSY NMR spectrum data of **4** at 800 MHz in pyridine-d_5.

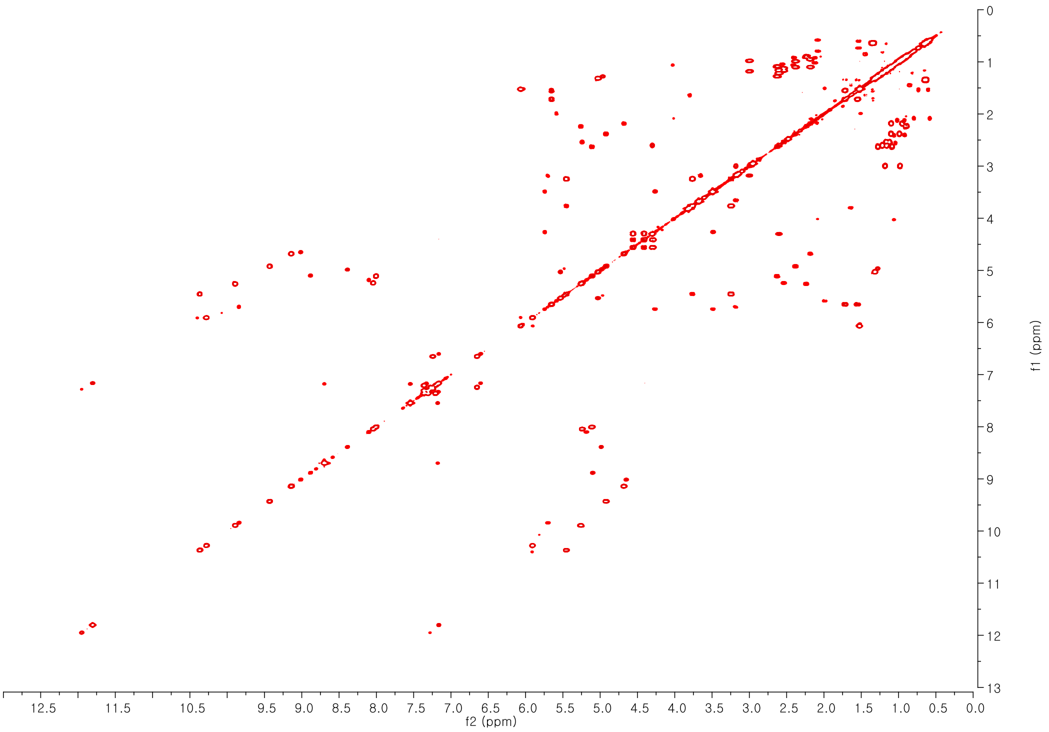

Figure S9. TOCSY NMR spectrum data of **4** at 800 MHz in pyridine-d_5.

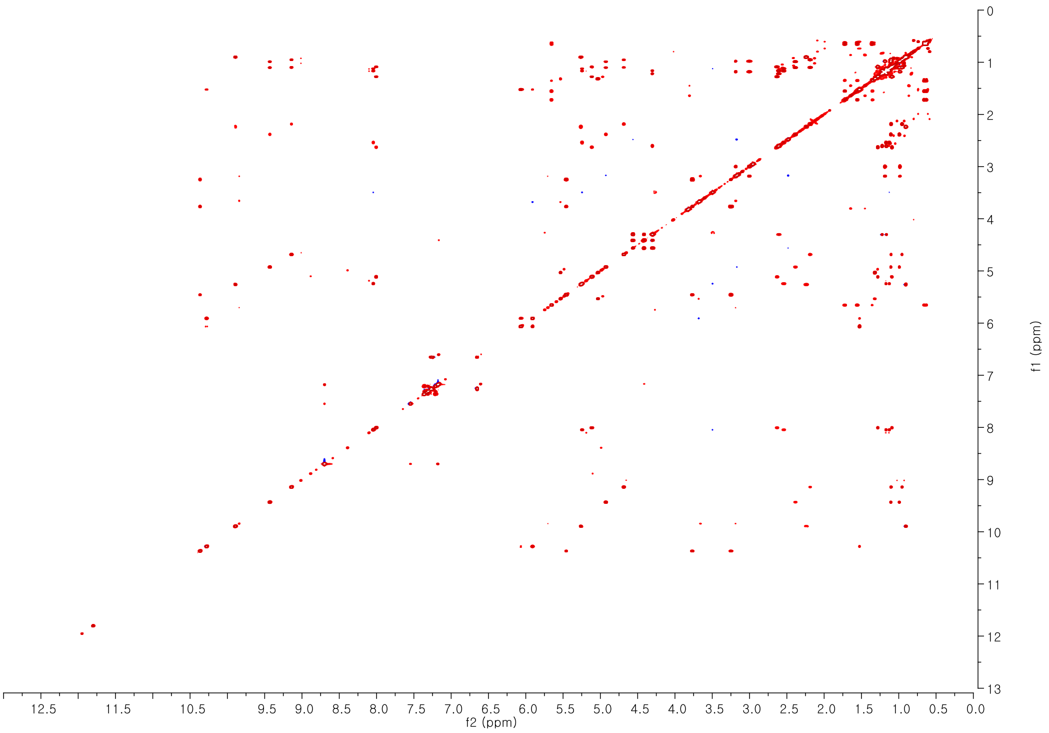

Figure S10. HSQC NMR spectrum data of **4** at 800 MHz in pyridine-*d*5.

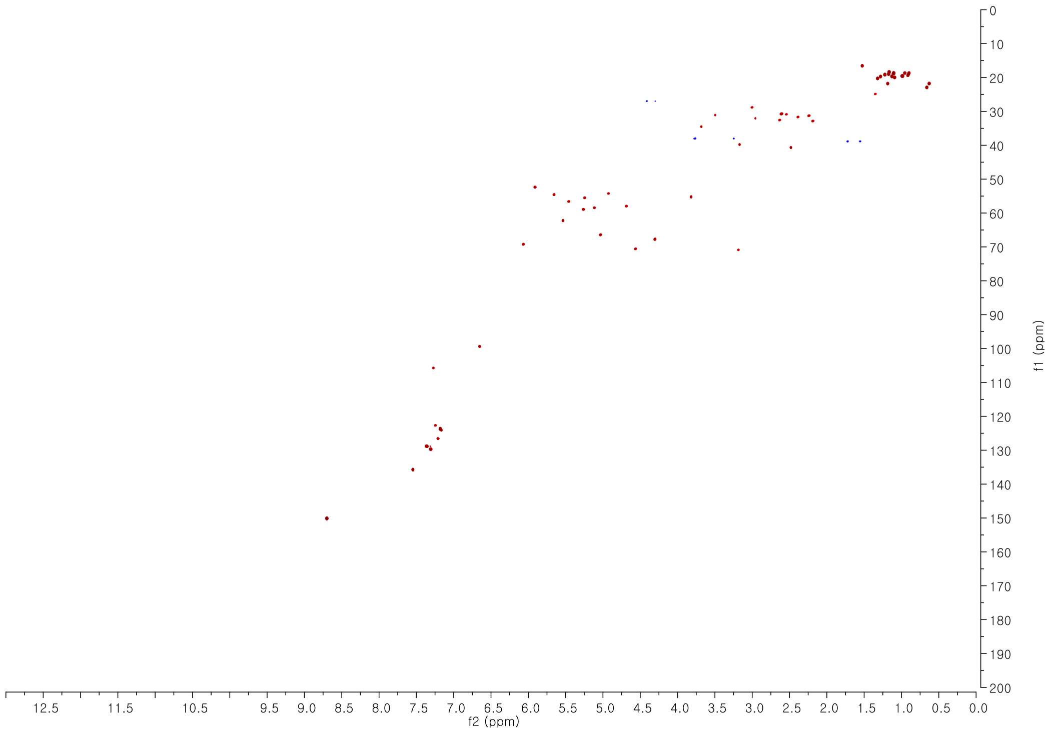

Figure S11. HMBC NMR spectrum data of **4** at 800 MHz in pyridine-*d*5.

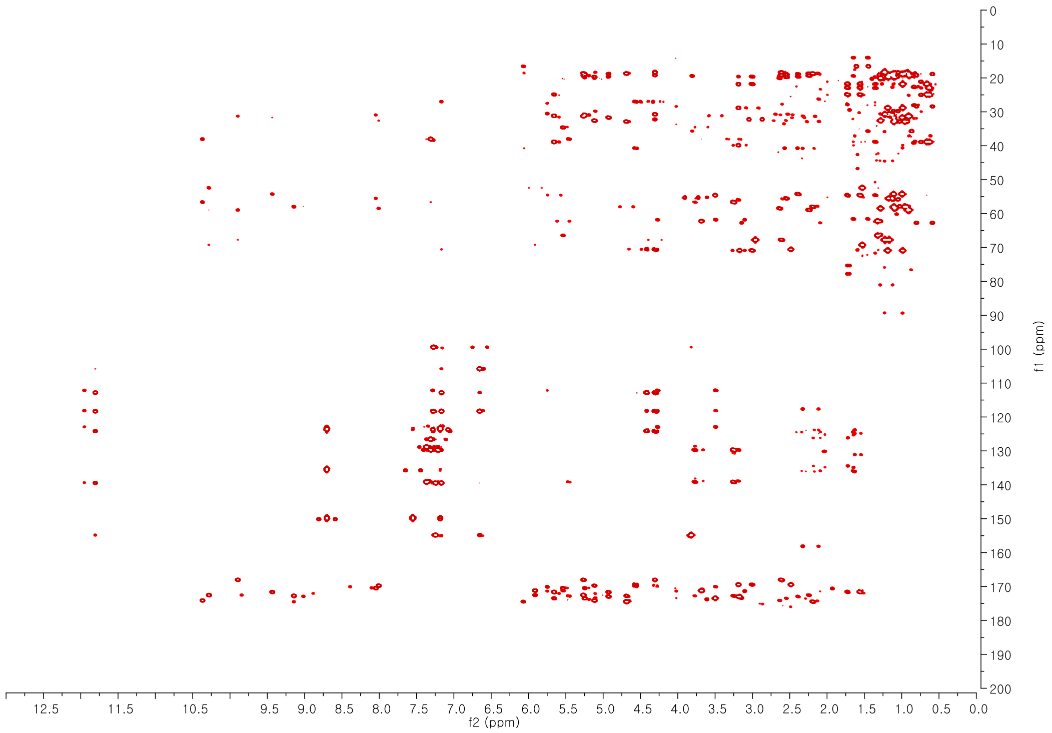

Figure S12. ROESY NMR spectrum data of **4** at 800 MHz in pyridine-d_5.

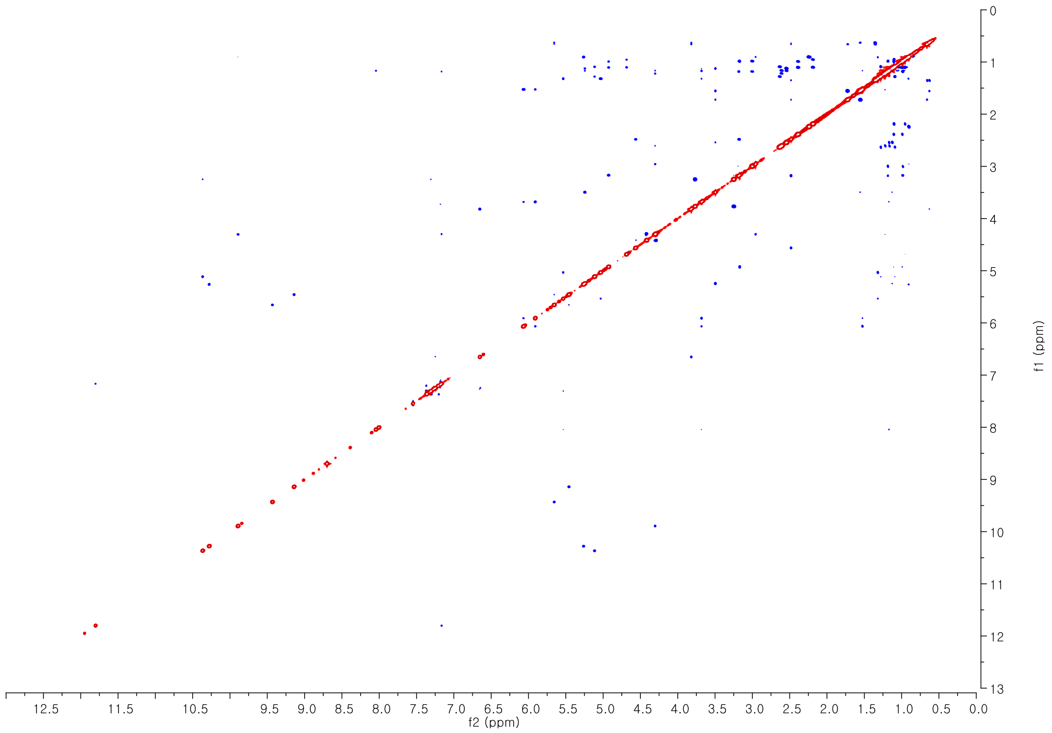

Figure S13. Comparing CD spectra data of **1**, **4**, and **6**.

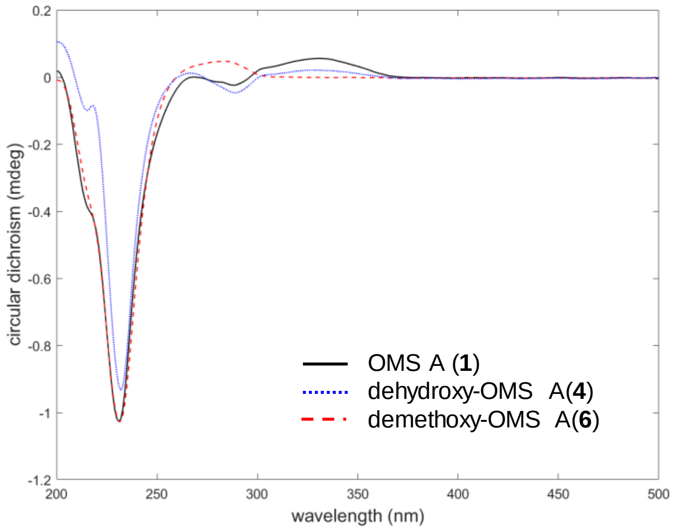

Figure S14. Phylogenetic relationships of known TDOs from secondary metabolite biosynthesis, including OhmK.

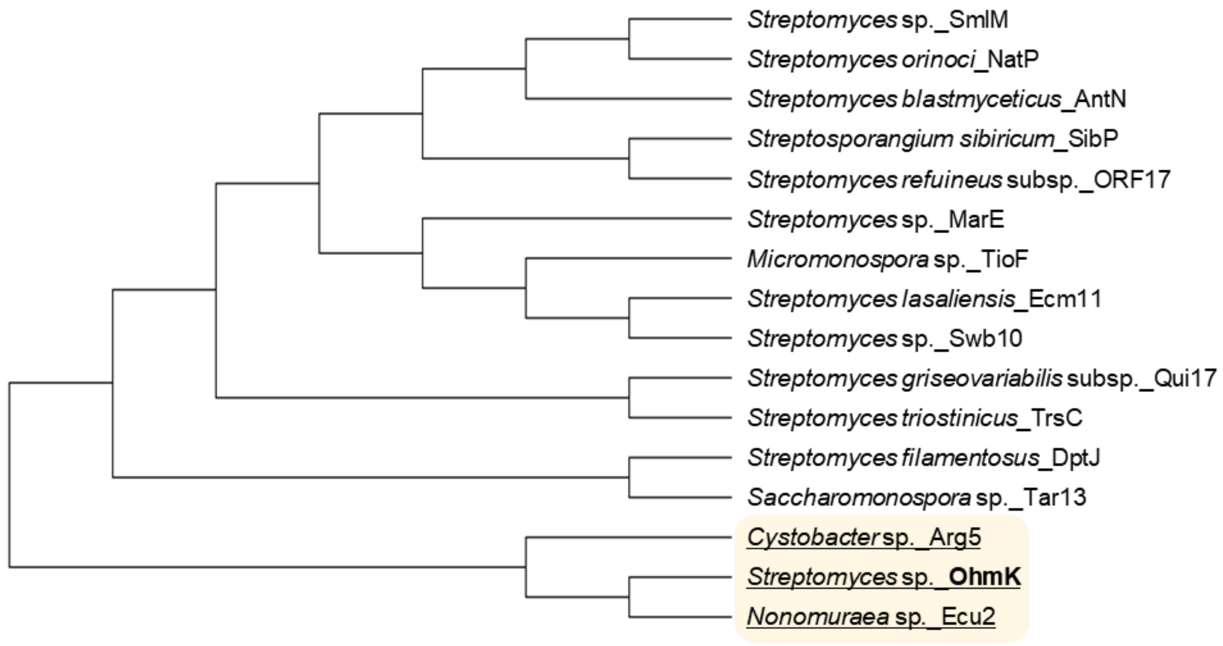

Accession numbers: SmlM (BBD17752.1, *Streptomyces* sp. ML55), NatP (BBD17773.1, *Streptomyces orinoci* NBRC 13466), AntN (AGG37776.1, *Streptomyces blastmyceticus* NBRC 12747), SibP (ACN39739.1, *Streptosporangium sibiricum* DSM 44039), ORF17 (ABW71848.1, *Streptomyces refuineus* subsp. thermotolerans), MarE (AHJ60974.1, *Streptomyces* sp. B9173), TioF (CAJ34362.1, *Micromonospora* sp. ML1), Ecm11 (BAE98160.1, *Streptomyces lasaliensis*), Swb10 (BAI63282.1, *Streptomyces* sp. SNA15896), Qui17 (AET98915.1, *Streptomyces griseovariabilis* subsp. bandungensis strain 2507), TrsC (BAH04172.1, *Streptomyces triostinicus*), DptJ (AAX31563.1, *Streptomyces filamentosus* NRRL 11379), Tar13 (AHH53511.1, *Saccharomonospora* sp. CNQ490), Arg5 (QCE43605.1, *Cystobacter* sp. SBCb004), Ecu2 (AIW58897.1, *Nonomuraea* sp. MJM5123)

Figure S15. HR-FAB-MS data of **6**.

[Elemental Composition] Page: 1

Data : FAB-S235 Date : 15-Mar-2019 21:24

Sample: 863.1427

Note : m-NBA

Inlet : Direct Ion Mode : FAB+

RT : 0.73 min Scan#: (34,35)

Elements : C 100/0, H 200/0, N 15/10, O 20/10

Mass Tolerance : 20ppm, 5mmu if m/z < 250, 10mmu if m/z > 500

Unsaturation (U.S.) : -0.5 - 50.0

Observed m/z	Int%	Err[ppm / mmu]	U.S.	Composition
1428.8860	56.8	-5.4 / -7.8	31.0	C 82 H 116 N 12 O 10
		+3.4 / +4.8	31.5	C 81 H 114 N 13 O 10
		+2.4 / +3.5	31.0	C 83 H 116 N 10 O 11
		-2.6 / -3.7	27.0	C 77 H 116 N 14 O 12
		+6.2 / +8.8	27.5	C 76 H 114 N 15 O 12
		-3.5 / -5.1	26.5	C 79 H 118 N 11 O 13
		+5.3 / +7.5	27.0	C 78 H 116 N 12 O 13
		-0.7 / -1.0	22.5	C 74 H 118 N 13 O 15
		-1.7 / -2.4	22.0	C 76 H 120 N 10 O 16
		-6.7 / -9.6	18.0	C 70 H 120 N 14 O 17
		+2.1 / +3.0	18.5	C 69 H 118 N 15 O 17
		+1.1 / +1.6	18.0	C 71 H 120 N 12 O 18
		-4.8 / -6.9	13.5	C 67 H 122 N 13 O 20
		+4.0 / +5.7	14.0	C 66 H 120 N 14 O 20

Figure S16. ^{1}H NMR spectrum data of **6** at 850 MHz in pyridine-d_5.

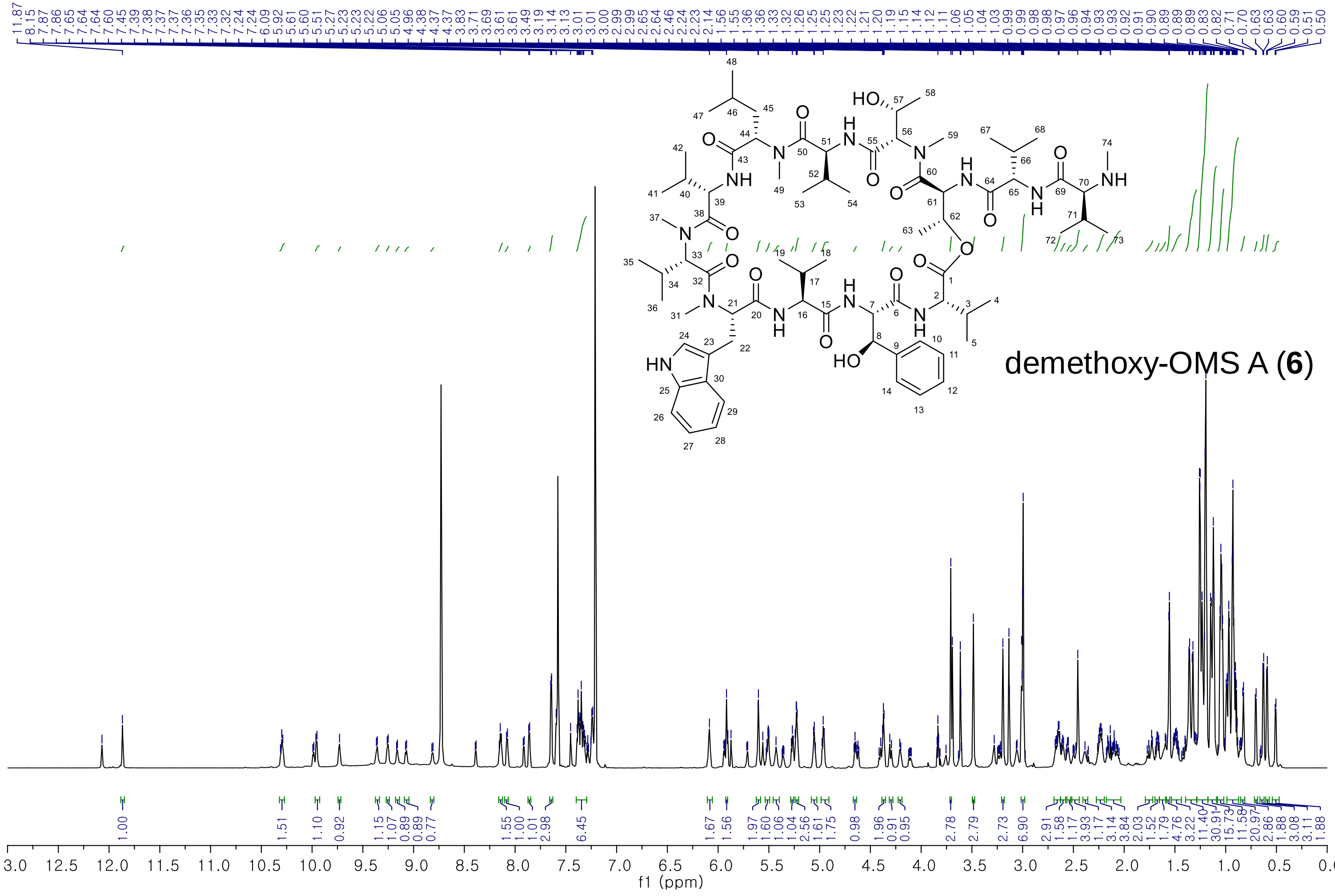

Figure S17. ^{13}C NMR spectrum data of **6** at 212.5 MHz in pyridine-d_5.

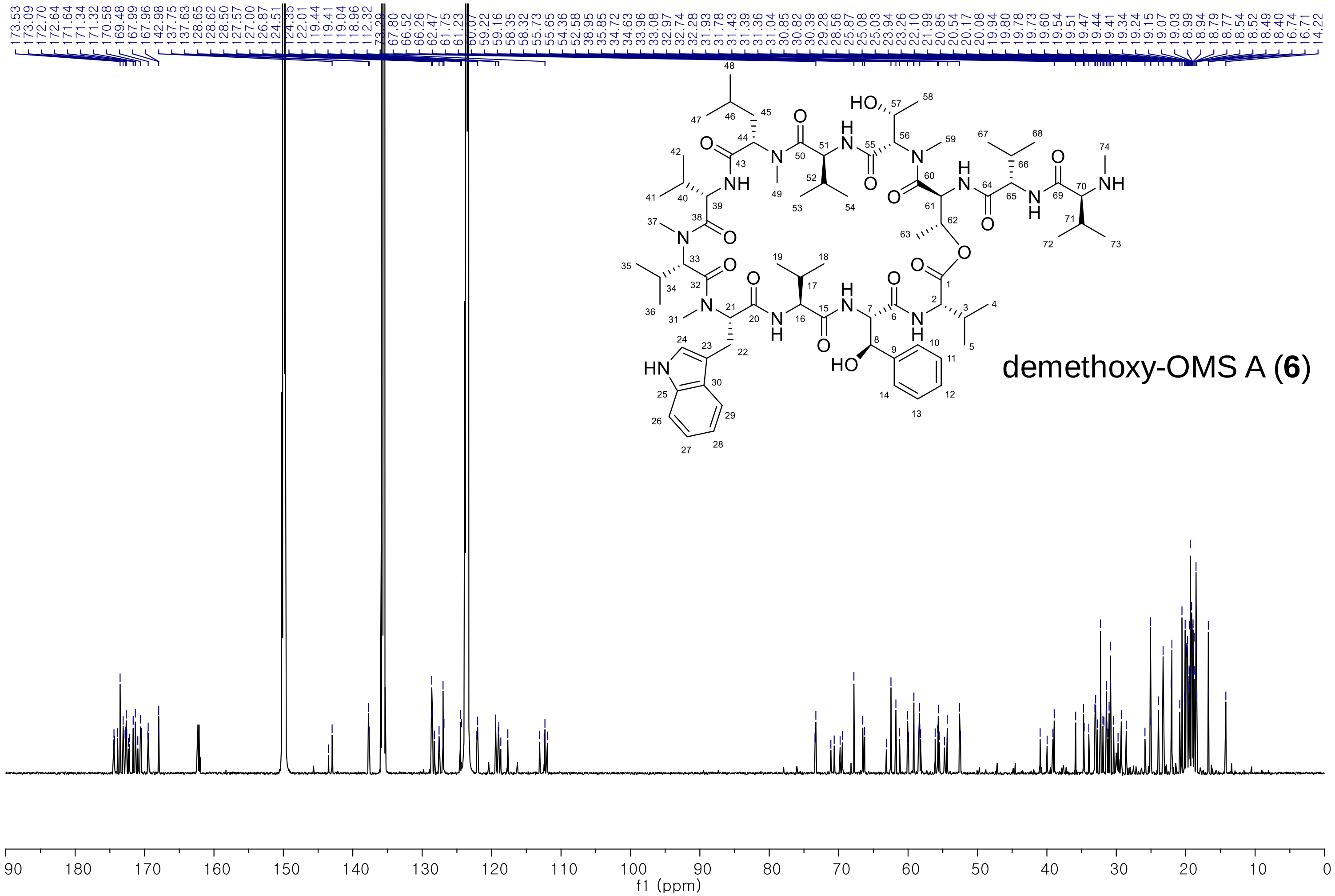

Figure S18. COSY NMR spectrum data of **6** at 850 MHz in pyridine-d_5.

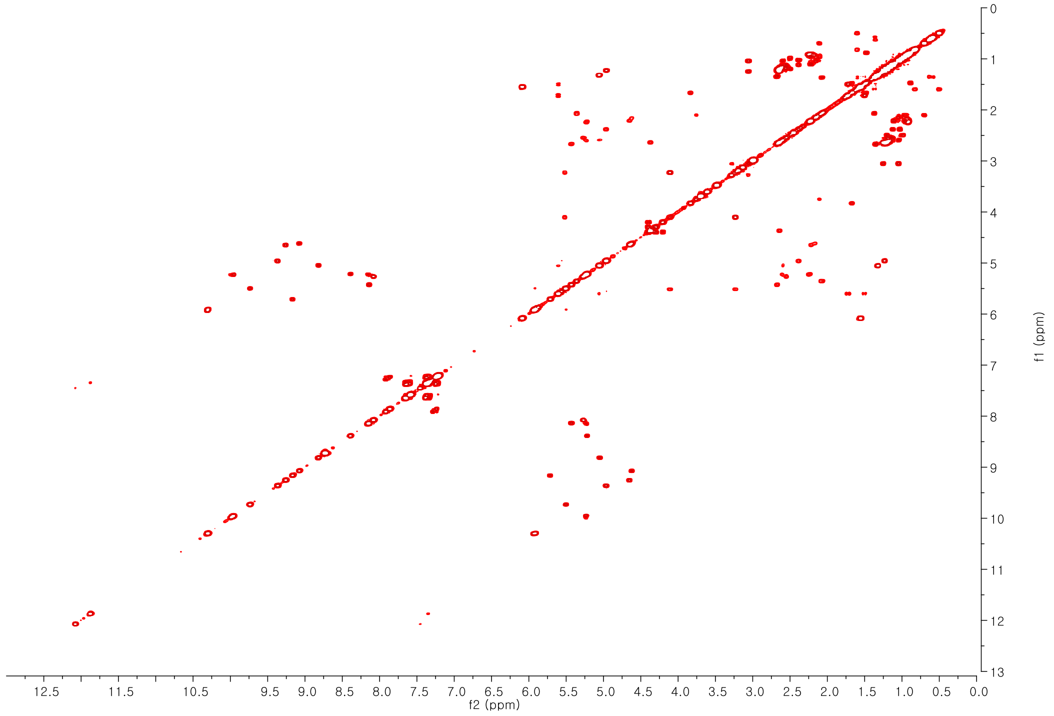

Figure S19. TOCSY NMR spectrum data of **6** at 850 MHz in pyridine-d_5.

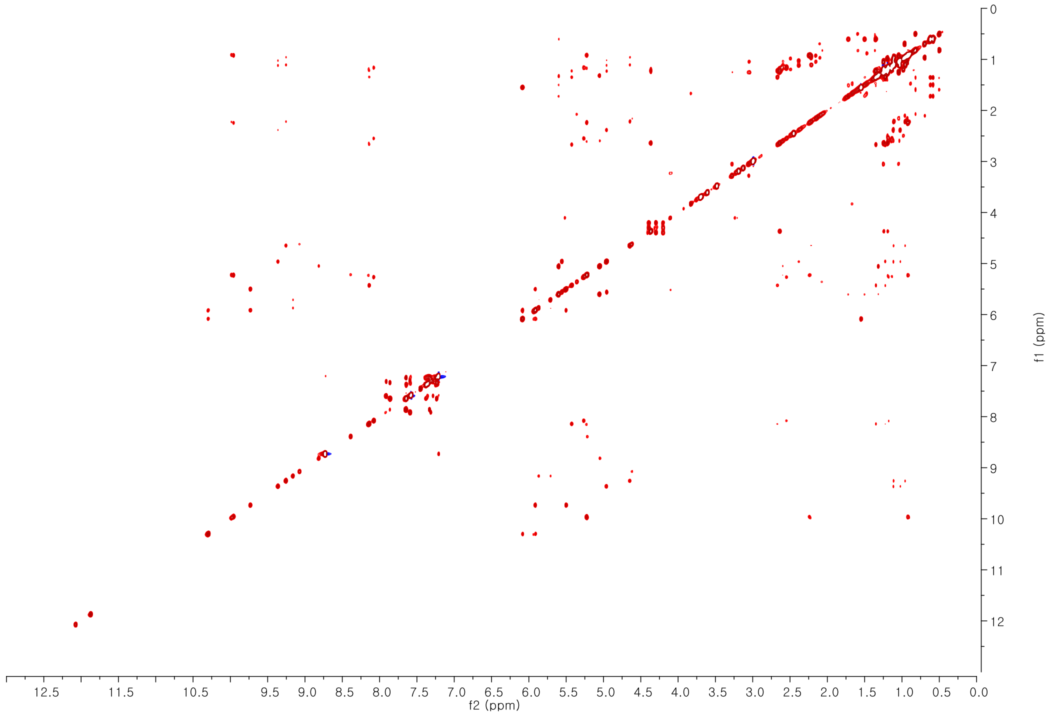

Figure S20. HSQC NMR spectrum data of **6** at 850 MHz in pyridine-*d*5.

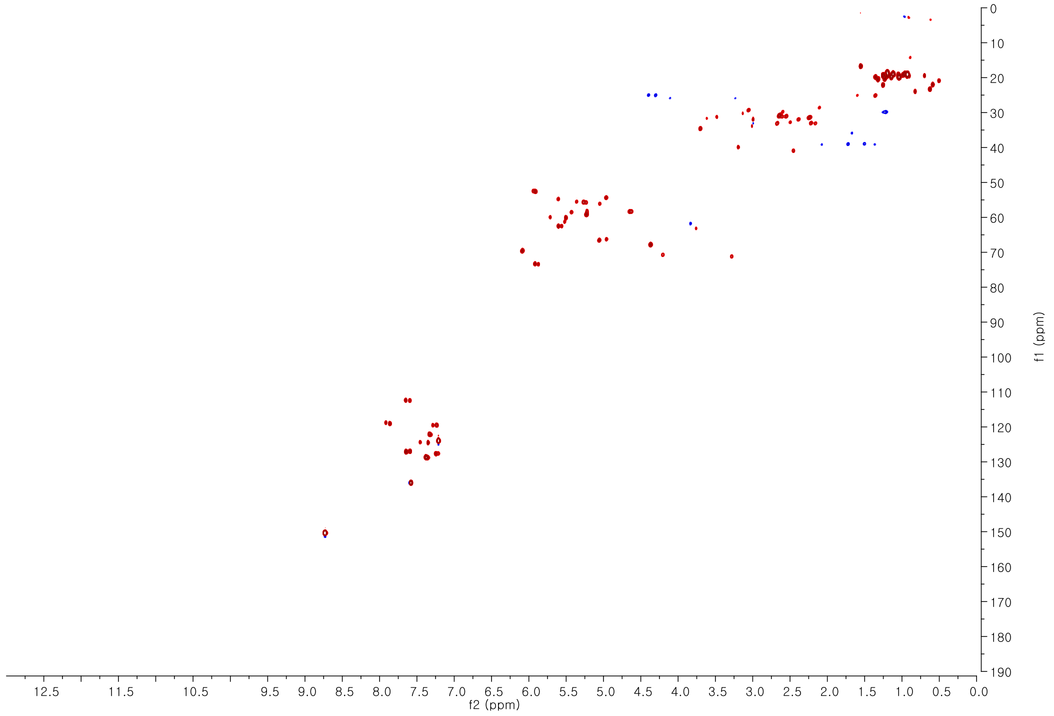

Figure S21. HMBC NMR spectrum data of **6** at 850 MHz in pyridine-*d*5.

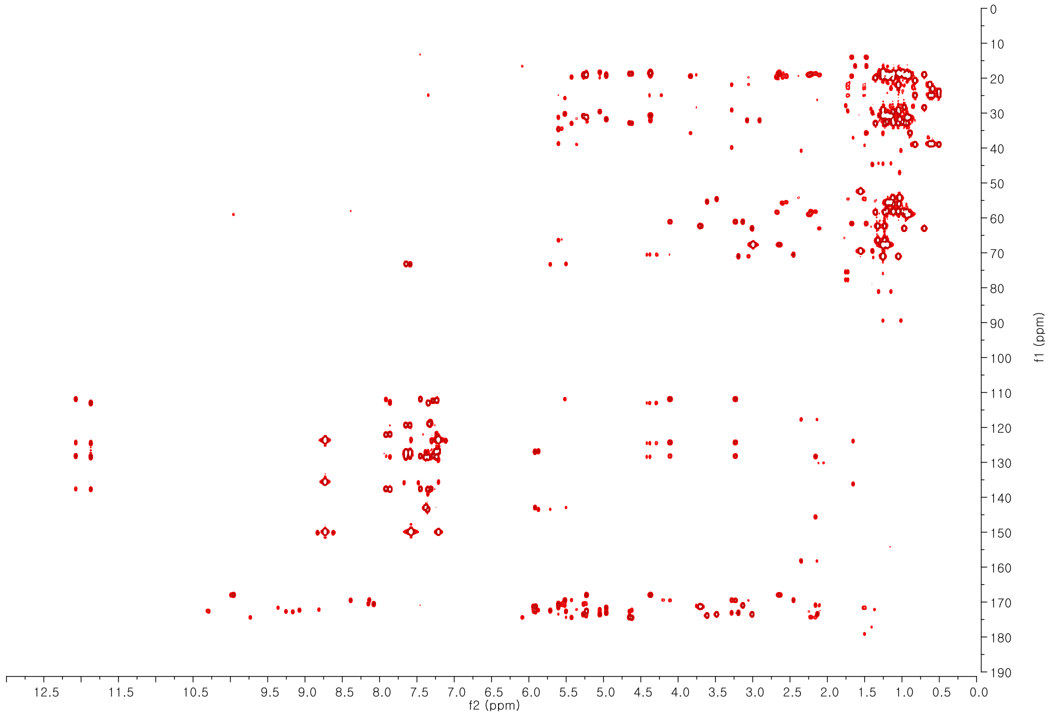

Figure S22. ROESY NMR spectrum data of **6** at 850 MHz in pyridine-d_5.

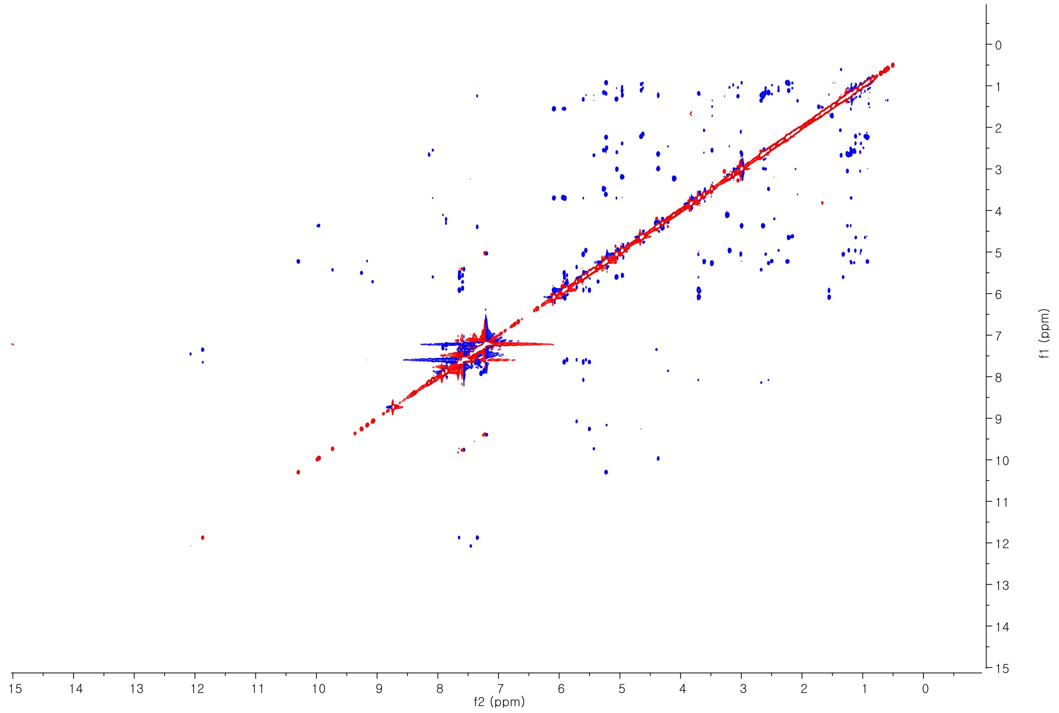

Table S1. Deduced function of ORFs in the ohmyungsamycin biosynthetic gene cluster.

ORF	Size (aa)	Proposed function	Homologue in *Nonomuraea* sp. MJM5123	
			Protein / GenBank (residues)	Identity/ Similarity
Orf(-2)	200	Hypothetical protein	-	-
Orf(-1)	291	Transposase	-	-
OhmA	13825	Nonribosomal peptide synthetase	Ecu11 / AIW58892.1 (15259)	64/74
OhmB	73	MbtH-like protein	Ecu10 / AIW58893.1 (72)	76/87
OhmC	78	Hypothetical protein	Ecu9 / AIW58894.1 (78)	77/84
OhmD	362	Putative ATPase AAA	Ecu8 / AIW58895.1 (362)	78/86
OhmE	436	Hypothetical protein	Ecu7 / AIW58902.1 (431)	78/86
OhmF	276	Hypothetical protein	Ecu6 / AIW58901.1 (233)	60/75
OhmG	400	Transposase	-	-
OhmH	320	ABC transporter ATP-binding protein	Ecu5 / AIW58900.1 (291)	72/84
OhmI	277	ABC-2 type transporter	Ecu4 / AIW58899.1 (276)	75/85
OhmJ	333	*O*-Methyltransferase	Ecu3 / AIW58898.1 (333)	61/73
OhmK	359	Putative tryptophan 2, 3-dioxygenase	Ecu2 / AIW58897.1 (368)	52/62
OhmL	398	Cytochrome P450 monooxygenase	Ecu1 / AIW58896.1 (447)	71/81
Orf(+1)	476	Hypothetical protein	-	-
Orf(+2)	327	Acetyl xylan esterase	-	-

Table S2. Substrate specificity sequences for adenylation (A) domains from ohmyungsamycin NRPS.

A domain	Substrate specific sequence	Predicted substrate	Postulated substrate
A1	D A Y W W G G T	Val	L-Val
A2	D A Y W G G A T	Val	L-Val/L-Ile
A3	D F W N V G M V	Thr	L-Thr
A4	D F W N I G M V	Thr	L-Thr
A5	D A Y W W G G T	Val	L-Val
A6	D A L L I G A I	Phe/Trp	L-Leu
A7	D A Y W W G G T	Val	L-Val
A8	D A Y W W G G T	Val	L-Val
A9	D V A L V G A V	Trp	4-MeO-L-Trp
A10	D A Y W W G G T	Val	L-Val
A11	D A W T V A A V	Phe	*β*-OH-L-Phe
A12	D A Y W W G G T	Val	L-Val

Table S3. Bacterial strains and plasmids used in this study.

Strain and plasmid	Description	Reference
**Strain**		
E. coli DH5α	Host for general cloning	New England Biolabs
E. coli ET12567/pUZ8002	Methylation-deficient donor strain for conjugal transfer between *E. coli* and *Streptomyces*	[1]
Streptomyces sp. SNJ042	Wild type ohmyungsamycin-producing strain	[2]
ΔohmL	The *ohmL* gene deleted mutant of SNJ042	This study
ΔohmK	The *ohmK* gene deleted mutant of SNJ042	This study
ΔohmJ	The *ohmJ* gene deleted mutant of SNJ042	This study
ΔohmK/ohmK	The *ohmK* gene complement mutant of *ΔohmK*	This study
ΔohmJ/ohmJ	The *ohmJ* gene complement mutant of *ΔohmJ*	This study
ΔohmJ/ohmK	The *ohmK* gene complement mutant of *ΔohmJ*	This study
**Plasmid**		
pKC1139	Temperature-sensitive *E. coli-Streptomyces* shuttle vector for gene disruption; *oriT* and Apr^R	[3]
pSET152	Integrative *E. coli-Streptomyces* shuttle vector containing P*ermE** for gene expression; *oriT*, *attP*, ΦC31 *int*, and Apr^R	[3]
pDel-OhmL	pKC1139 derivative for in-frame deletion of *ohmL* gene	This study
pDel-OhmK	pKC1139 derivative for in-frame deletion of *ohmK* gene	This study
pDel-OhmJ	pKC1139 derivative for in-frame deletion of *ohmJ* gene	This study
pOhmK	pSET152 derivative for expression of *ohmK* gene	This study
pOhmJ	pSET152 derivative for expression of *ohmJ* gene	This study

Table S4. Primers used in this study.

Gene	Primer	Sequences (5'-3')	Restriction site
**In-frame deletion**			
ohmL (P450)	delL_LF	AAAGAATTCGCGATGTGGCAGCTCAGTTT	*Eco*RI
	delL_LR	AATCTAGATGAGCGGGCGGAACGGCGCG	*Xba*I
	delL_RF	AAATCTAGAGACCACGTGATCGCTCCCCT	*Xba*I
	delL_RR	TTAAGCTTGATCTATCTGCTCGCCTTCC	*Hind*III
ohmK (TDO)	delK_LF	AATTGAATTCATGGTGAACTCGCTCCTCGA	*Eco*RI
	delK_LR	AATCTAGACACATTCCCCCTGGAACGTG	*Xba*I
	delK_RF	TTAATCTAGAGTGCACTTCGCCTACCGCTG	*Xba*I
	delK_RR	TTAAGCTTGCTGAGCGGGAGATTCCTCC	*Hind*III
ohmJ (O-MT)	delJ_LF	AATCAATTGAGTGCAGAACCTCCAGATGT	*Mfe*I
	delJ_LR	AAATCTAGACATTCTCCACCCCCAGTGAA	*Xba*I
	delJ_RF	AATCTAGATGACACGTTCCAGGGGGAAT	*Xba*I
	delJ_RR	TTAAGCTTACGAGGAGATCTTCCTCAAC	*Hind*III
**Complementation**			
ohmK (TDO)	ohmK_F	AATTAGATCTTGACACGTTCCAGGGGGAAT	*Bgl*II
	ohmK_R	AATCTAGAGCCTCAGCGGTAGGCGAAGT	*Xba*I
ohmJ (O-MT)	ohmJ_F	AATTAGATCTTCGCTCGGGTAGTGTCACCT	*Bgl*II
	ohmJ_R	AATCTAGAGTGTCAGGGCTTGTGGGCCA	*Xba*I

Table S5. ^{1}H NMR data for minor conformer of **4** in pyridine-*d*$_5$.

	position	type	δ_H		position	type	δ_H
Val-1	1	C		Val-6	38	C	
	2	CH	4.69		39	CH	5.13
	2-NH		9.05		39-NH		8.91
	3	CH	2.15		40	CH	2.59
	4	CH_3	1.06		41	CH_3	1.16
	5	CH_3	0.95		42	CH_3	1.08
Phe-2	6	C		*N*-Me-	43	C	
	7	CH	5.73	Leu-7	44	CH	5.62
	7-NH		9.87		45a	CH_2	2.02
	8a	CH_2	3.68		45b		1.54
	8b		3.20		46	CH	1.56
	9	C			47	CH_3	0.77
	10	CH	7.30		48	CH_3	0.64
	11	CH	7.37		49	CH_3	3.64
	12	CH	7.21	Val-8	50	C	
	13	CH	7.37		51	CH	5.22
	14	CH	7.30		51-NH		8.13
Val-3	15	C			52	CH	2.62
	16	CH	5.02		53	CH_3	1.25
	16-NH		8.41		54	CH_3	1.14
	17	CH	2.44	*N*-Me-	55	C	
	18	CH_3	1.10	Thr-9	56	CH	5.52
	19	CH_3	0.96		57	CH	5.00
N-Me-4-	20	C			58	CH_3	1.30
methoxy-	21	CH	5.77		59	CH_3	3.70
Trp-4	22a	CH_2	4.30	Thr-10	60	C	
	22b		3.51		61	CH	5.93
	23	C			61-NH		10.30
	24	CH	7.31		62	CH	6.11
	24-NH		11.97		63	CH_3	1.55
	25	C		Val-11	64	C	
	26	CH	7.36		65	CH	5.28
	27	CH	7.20		65-NH		9.93
	28	CH	6.64		66	CH	2.27
	29	C			67	CH_3	0.94
	29-OMe	CH_3	3.87		68	CH_3	0.93
	30	C		*N*-Me-	69	C	
	31	CH_3	3.13	Val-12	70	CH	4.33
N-Me-	32	C			71	CH	2.64
Val-5	33	CH	4.04		72	CH_3	1.24
	34	CH	2.11		73	CH_3	1.18
	35	CH_3	0.83		74	CH_3	2.98
	36	CH_3	0.61				
	37	CH_3	3.17				

^{1}H NMR data were measured at 800 MHz.

Figure S23. Key ROESY correlations of (A) major and (B) minor conformers of dehydroxy-OMS A (**4**).

A

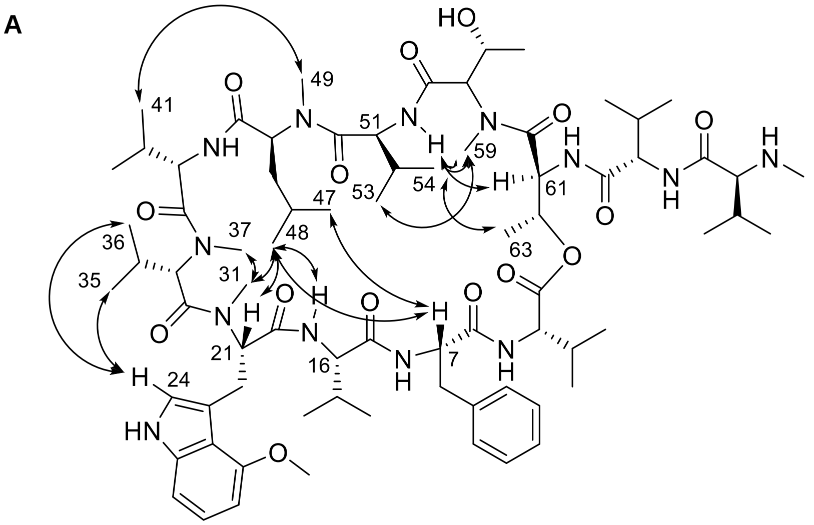

B

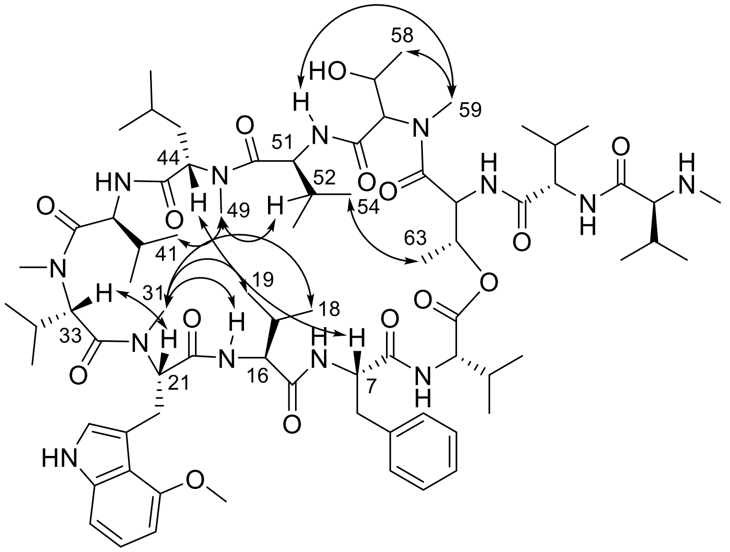

Table S6. ^{1}H NMR data for minor conformer of **6** in pyridine-*d*$_5$.

	position	type	δ_H		position	type	δ_H
Val-1	1	C		Val-6	38	C	
	2	CH	4.62		39	CH	5.05
	2-NH		9.08		39-NH		8.81
	3	CH	2.17		40	CH	2.59
	4	CH_3	0.99		41	CH_3	1.14
	5	CH_3	1.05		42	CH_3	1.04
β-OH-	6	C		*N*-Me-	43	C	
Phe-2	7	CH	5.71	Leu-7	44	CH	5.36
	7-NH		9.16		45a	CH_2	2.07
	8	CH	5.88		45b		1.39
	9	C			46	CH	1.60
	10	CH	7.59		47	CH_3	0.83
	11	CH	7.37		48	CH_3	0.50
	12	CH	7.25		49	CH_3	3.61
	13	CH	7.37	Val-8	50	C	
	14	CH	7.59		51	CH	5.22
Val-3	15	C			51-NH		8.39
	16	CH	5.23		52	CH	2.50
	16-NH		8.16		53	CH_3	1.20
	17	CH	2.23		54	CH_3	0.99
	18	CH_3	0.93	*N*-Me-	55	C	
	19	CH_3	0.93	Thr-9	56	CH	5.56
N-Me-	20	C			57	CH	4.95
Trp-4	21	CH	5.52		58	CH_3	1.25
	22a	CH_2	4.11		59	CH_3	3.69
	22b		3.23	Thr-10	60	C	
	23	C			61	CH	5.94
	24	CH	7.45		61-NH		10.30
	24-NH		12.07		62	CH	6.09
	25	C			63	CH_3	1.56
	26	CH	7.59	Val-11	64	C	
	27	CH	7.37		65	CH	5.24
	28	CH	7.29		65-NH		9.98
	29	CH	7.91		66	CH	2.24
	30	C			67	CH_3	0.92
	31	CH_3	3.14		68	CH_3	0.92
N-Me-	32	C		N-Me-	69	C	
Val-5	33	CH	3.76	Val-12	70	CH	4.37
	34	CH	2.10		71	CH	2.65
	35	CH_3	0.98		72	CH_3	1.25
	36	CH_3	0.71		73	CH_3	1.20
	37	CH_3	3.01		74	CH_3	3.00

^{1}H NMR data were measured at 850 MHz.

Figure S24. Key ROESY correlations of (A) major and (B) minor conformers of demethoxy-OMS A (**6**).

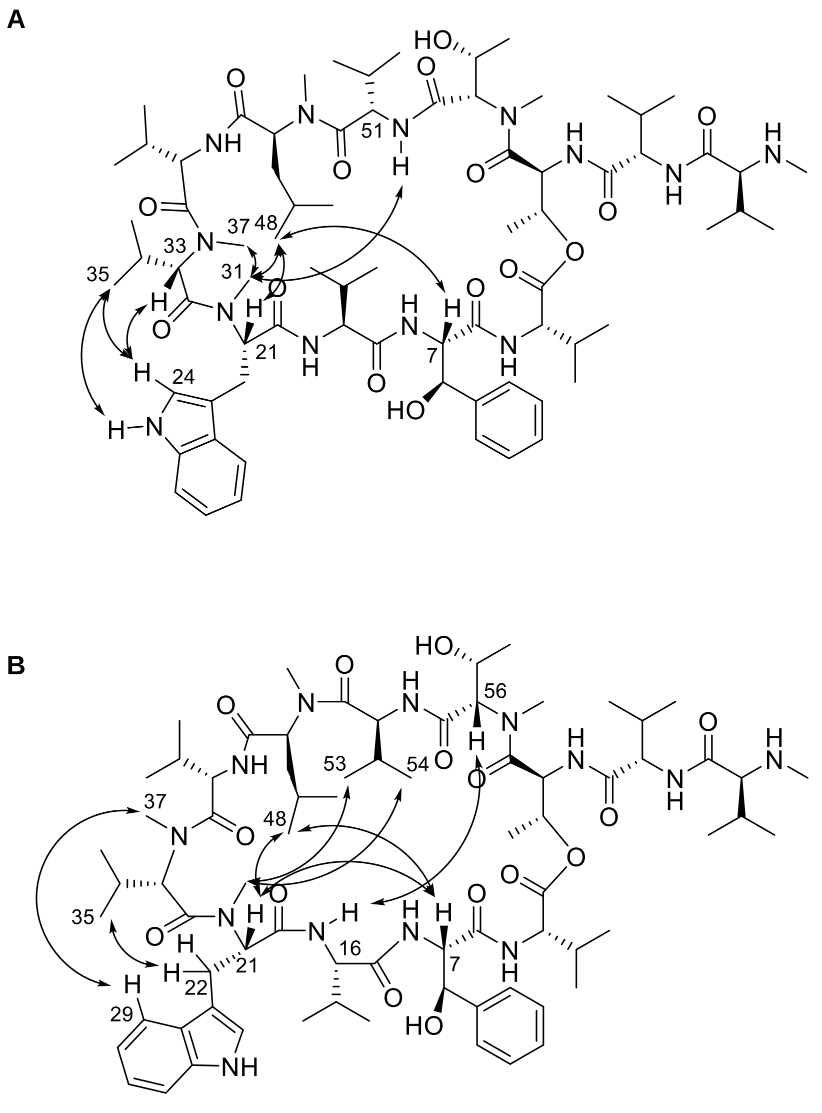

References

1. Kieser, T.; Bibb, M. J.; Buttner, M. J.; Chater, K. F.; Hoopwood, D. A. *Practical Streptomyces Genetics*; The John Innes Foundation: Norwich, UK, 2000.
2. Um, S.; Choi, T. J.; Kim, H.; Kim, B. Y.; Kim, S.-H.; Lee, S. K.; Oh, K.-B.; Shin, J.; Oh, D.-C. Ohmyungsamycins A and B: cytotoxic and antimicrobial cyclic peptides produced by *Streptomyces* sp. from a volcanic island. *J. Org. Chem.* **2013**, *78*, 12321-12329.
3. Bierman, M.; Logan, R.; O'Brien, K.; Seno, E. T.; Rao, R. N.; Schoner, B. E. Plasmid cloning vectors for the conjugal transfer of DNA from *Escherichia coli* to *Streptomyces* spp. *Gene* **1992**, 116, 43-49.
